# Extensive Genome-Wide Variability of Human Cytomegalovirus in Congenitally Infected Infants

**DOI:** 10.1371/journal.ppat.1001344

**Published:** 2011-05-19

**Authors:** Nicholas Renzette, Bornali Bhattacharjee, Jeffrey D. Jensen, Laura Gibson, Timothy F. Kowalik

**Affiliations:** 1 Department of Microbiology and Physiological Systems, University of Massachusetts Medical School, Worcester, Massachusetts, United States of America; 2 Program in Bioinformatics & Integrative Biology, University of Massachusetts Medical School, Worcester, Massachusetts, United States of America; 3 Departments of Pediatrics and Medicine, Divisions of Infectious Diseases and Immunology, University of Massachusetts Medical School, Worcester, Massachusetts, United States of America; 4 Immunology and Virology Program, University of Massachusetts Medical School, Worcester, Massachusetts, United States of America; University of Wisconsin-Madison, United States of America

## Abstract

Research has shown that RNA virus populations are highly variable, most likely due to low fidelity replication of RNA genomes. It is generally assumed that populations of DNA viruses will be less complex and show reduced variability when compared to RNA viruses. Here, we describe the use of high throughput sequencing for a genome wide study of viral populations from urine samples of neonates with congenital human cytomegalovirus (HCMV) infections. We show that HCMV intrahost genomic variability, both at the nucleotide and amino acid level, is comparable to many RNA viruses, including HIV. Within intrahost populations, we find evidence of selective sweeps that may have resulted from immune-mediated mechanisms. Similarly, genome wide, population genetic analyses suggest that positive selection has contributed to the divergence of the HCMV species from its most recent ancestor. These data provide evidence that HCMV, a virus with a large dsDNA genome, exists as a complex mixture of genome types in humans and offer insights into the evolution of the virus.

## Author Summary

Human Cytomegalovirus (HCMV) is a dsDNA virus that is the leading source of birth defects associated with an infectious agent. There is currently no effective HCMV vaccine and few treatment strategies for congenital infections exist. Thus, a better understanding of HCMV infections is warranted. Limited data has shown that HCMV exists as a mixture of a few genotypes in human hosts. Here, we describe our use of high throughput sequencing to study the extent of genome wide variability within HCMV infections sampled from congenital infections. Surprisingly, we find that HCMV populations are as variable as quasispecies RNA viruses; it is commonly believed that DNA viruses are more genetically stable than RNA viruses, and thus produce homogenous populations. Additionally, we find evidence of evolutionary pressures acting on the HCMV genome, both within and among populations. These results provide the first evidence that diversity of DNA virus populations can be comparable to that of RNA virus populations.

## Introduction

Human cytomegalovirus (HCMV) is member of the β-herpesvirus family. It is a ubiquitous, opportunistic pathogen, with seroprevalence of 30–90% in the United States [1]. In healthy individuals, primary HCMV infection is usually asymptomatic or can result in a mild febrile illness. However, infection persists throughout the life of the host. HCMV infections can be problematic for those with compromised or immature immune systems. For example, congenital HCMV infection is the leading cause of birth defects resulting from an infectious agent, affecting about 0.5% of all live births [2] and costing the U.S. Health care system ∼$2 billion annually [3]. Long term sequelae of congenital HCMV infections include deafness, blindness and/or mental disability [4].

HCMV contains the largest genome of any human virus with a dsDNA genome of ∼236 kilobase pairs [5]. Sequence analysis predicts that the genome encodes approximately 164 open readings frames (ORFs) [6]. The genome contains two unique regions (termed U_L_ and U_S_) that are flanked by repeats (termed R_L_ and R_S_) both internally and terminally, although the internal R_L_ region is not present in clinical isolates or low passage strains. Previous work with cell culture passed virus has shown that the genome of HCMV displays sequence variability. For example, the laboratory strain AD169 is a highly passaged, attenuated variant. The genome of AD169 as compared to low passage strains has an approximately 15 kb deletion which encodes an additional 19 or 22 open ORFs, referred to as the UL/b' region [6], [7], [8]. Approximately 20 ORFs of HCMV have been shown to exhibit nucleotide variability when sequenced from infected hosts [9], [10], [11], [12], [13], [14], [15], [16], [17], [18], [19]. These studies have often focused on the variability of ORFs encoding envelope glycoproteins or ORFs of UL/b', which are thought to be important for pathogenesis. As examples, *UL55* and *UL73*, encoding the gB and gN glycoproteins, respectively, commonly exist as one of 4 genotypes, with less common genotypes also identified [19], [20]. In the UL/b' region, *UL144*, encoding a TNF-α receptor [21], and *UL146* and *UL147*, encoding α-chemokines [22], also show significant variability among hosts [9], [14], [23], [24], [25].

Although it is known that HCMV is polymorphic among hosts, the source of the variability remains unresolved. There are at least two possibilities to explain the observation. The first is that *de novo* mutations arise upon introduction into a new host, resulting in a unique strain for each individual. The second possibility is that multiple HCMV genotypes exist within each host, and infection into a new host represents a selection event whereby a new dominant genotype is selected for and detected in subsequent assays. In support of this model, others have found evidence of mixed genotype populations at the few loci examined. Mixed populations have been observed when measuring *gB* genotypes [26], [27], [28], [29], [30], though the phenomenon has also been shown for other ORFs, such as *gN*, *gO*, *gH*, *gL*, *UL139*, and *UL146* [31], [32], [33], [34], [35], [36], [37]. Furthermore, mixed populations have been shown in a range of patient populations, including immunocompetent, asymptomatic adults [31] and have been shown at multiple loci simultaneously [33]. While definitive relationships between genotypes and diseases are lacking, there is mounting evidence that mixed genotype infections serve as markers of severe or prolonged complications from HCMV infections [26], [27], [29], [30], [34], [36]. A shortcoming of the mixed genotype studies has been limited coverage of the HCMV genome. To our knowledge, less than 5% of the HCMV genome has been sequenced from clinical specimens in these types of studies (Figure S1). Thus, a remaining question is whether HCMV diversity is limited to a subset of ORFs or is found throughout the genome.

From earlier studies, it appears that HCMV may exist as a mixture of genotypes. Due to limitations of previous technology, it was unrealistic to study mixed HCMV populations to great depth or sequence the HCMV genome to high coverage. To address these shortcomings, we have adapted high throughput sequencing to sample many members of the HCMV genomic population, rather than just a dominant member. With the improved output of next generation sequencing, we were able to take a genome wide approach and sequence thousands of HCMV genome equivalents from each patient sample. Here we sampled the HCMV genomic populations present in urine samples collected from three congenitally infected newborns. These data reveal a high level of intrahost variability and offer strong evidence that HCMV exists as a complex mixture of variants. We also found evidence of selection at both the intrahost and interhost levels, highlighting evolutionary forces that shape the HCMV genome. These results greatly improve our understanding of the structure of HCMV populations in humans, and have important implications for the study of DNA viruses.

## Results

### Development of sequence methodology and error filtering protocol

In clinical samples, HCMV DNA represents a very low proportion of the total DNA. Thus, direct sequencing would yield a low depth of the HCMV population with human DNA being a major source of contaminant. Because there is homology between the human and HCMV genomes [38], [39], this contaminant would be problematic in downstream sequence analyses. We developed a series of approximately 70 long range, overlapping PCR reactions to selectively amplify the entire HCMV genome. However, PCR amplification can introduce errors of its own, which could be misinterpreted as polymorphisms. To assess the error associated with sample processing, we resequenced BACs that contained the genomes of the HCMV strains AD169 and Toledo. The BACs have been shotgun sequenced to a 10X depth [40], producing reliable reference sequences for these purposes.

The BAC DNA was amplified through a series of PCR reactions and sequenced on the Illumina GA II paired end platform. The sequence output was equivalent to ∼220 genomes per strain (Table 1). The sequence reads were aligned to the appropriate reference sequence and the alignments were analyzed for errors. We assumed that all mismatches between the sequencing reads and the reference sequence were errors introduced by either PCR or sequencing. This assumption is most likely conservative because there is the possibility that variants were created by propagating the BACs in *E. coli* or that errors could be present in the reference sequences. The alignment data contained in the pileup file was then processed with a variant filtering program. The variant filtering program only outputs variants that are above threshold values for basecall quality, mapping quality, depth at the position, number of occurrences of the same variant and frequency of the variant in the data. The thresholds used were: basecall quality ≥30, mapping quality ≥89, depth ≥15, number of occurrences ≥3, and frequency ≥.019. The basecall quality and mapping quality values are used to filter nucleotides with low confidence from sequencing or from reads that align with low confidence, respectively. Depth, number of occurrences of variant and frequency are used to remove likely errors because random errors (from either sample amplification or sequencing) have the highest likelihood of occurring as singletons and doubletons (1 or 2 occurrences). These threshold values were chosen by training the filtering program with BAC resequencing data. The resequencing data from AD169 and Toledo were mixed in various ratios to model a mixed population. The filtering thresholds were selected to increase specificity of detecting true variants; however, they carry a penalty of reducing sensitivity and underestimating the amount of variants in the sample (Table S1). The number of false positives remains low at various depths and mixtures of the sequences. We did not find evidence of amplification-induced skewing of variant frequencies. Further discussion of analysis of error can be found under Materials and Methods and Supplemental Information.

**Table 1:** Sequence output of high throughput sequencing experiments.

Source^1^	Type	Reads	% Aligned Reads	Sequence Output (Mb)	Depth^2^	Coverage^3^
AD169	BAC	774,803	94.9%	51.1	218	98.7%
Toledo	BAC	720,120	96.0%	47.5	226	96.8%
U01	Urine	2,444,677	74.9%	337.4	1493	97.0%
U04	Urine	3,395,157	74.7%	468.5	1990	98.7%
U33	Urine	3,490,699	80.7%	481.7	2046	97.7%

1 Source was BAC DNA encoding the AD169 or Toledo genome, or urine samples collected from neonates (<2 weeks from birth) with congenital HCMV infections.

2 Depth is the average number of reads that cover each position of the genome.

3 Coverage is expressed as percentage of the genome for which sequence data was generated. For AD169 and Toledo resequencing, the published sequence was used to calculate coverage. For clinical material sequencing, the coverage is estimated by using the Merlin strain reference genome.

### High throughput sequencing of clinical populations

We sampled HCMV genomic populations present in the urine collected from 3 HCMV-positive neonates within 2 weeks of birth (identified as U01, U04, and U33). The entire HCMV genome was amplified as discussed above. The PCR reactions and amount of template DNA were identical between the BAC resequencing and the clinical sequencing. Therefore, the error filtering protocol developed through BAC resequencing can be applied to the clinical sequence data. From clinical sequencing, >300 megabases of output per sample yielded an average depth of 1843 genome equivalents and an average genome coverage of 97.8% for the 3 samples (Table 1 and Figure S3).

**Figure 1:**
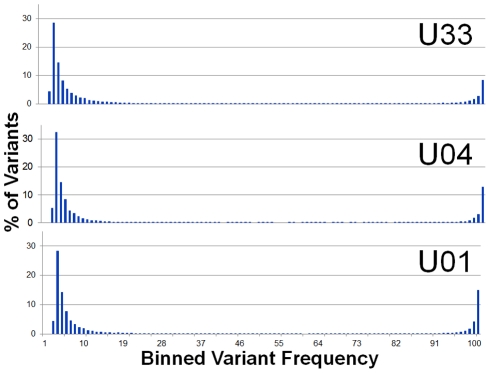
Single nucleotide variant frequencies of HCMV populations segregate into low and high frequency classes. Histogram of binned variant frequencies within three HCMV populations sampled from urine samples. The graphs are labeled according to patient sample (U01, U04, and U33). The variants have been filtered to reduce errors, thereby eliminating variants of frequency <1.9%. See Figure S2 for analysis of the effectiveness of the error filtering algorithm.

Initially, the sequence reads from the urine samples were aligned to the sequence of the Merlin strain, which was used as the HCMV reference genome (Ref Seq ID: NC_006273). From the alignment, >10^4^ single nucleotide variants (range: 11289–15709) were detected per viral population. Variants segregated into clusters at frequencies ≤.1 or ≥.9 (Figure 1). Variants with frequency ≤.1 represent on average 73% (Range 67%–78%) of the total and variants with frequency ≥.9 represent 20% (Range: 16%–24%). From these data, we conclude that the high frequency variants result from the major alleles found in the viral population while the low frequency variants result from the minor alleles.

### Generation of a sample specific genome type

To study HCMV intrahost variability, we defined the major HCMV genome type of each sample and called intrahost variants from this reference genome type (Figure S4). A genome type is the genome wide analog of a genotype [41], [42], [43]. The major genome type contains the major allele found at every position of the genome. Thus, any variants from this genome type represent minor alleles or minor variants. It should be noted that the genome type may not represent any single DNA molecule in the viral population. Rather, the major genome type is a computational tool that allows for the detection of minor variants in the population, and every position of the genome in this analysis and all later analyses are treated independently (i.e. unlinked).

To define the major genome type, output from an initial alignment to Merlin was used to detect variants with frequencies >0.5 (Figure S4). These variants were interpreted to represent the major allele of the sample at each position. Variants were incorporated into the reference sequence to create an initial sample-specific genome type. Reads that did not initially align were used as substrate for *de novo* contiguous sequence (contigs) assembly. The contigs were aligned to the initial sample-specific genome type and incorporated into the genome if sequence identity was found. This modified genome type was used to serve as the reference sequence for another round of alignment of the sequencing reads and subsequent incorporation of high frequency variants and assembly of contigs onto the sample specific genome type. This process of constructing a sample-specific genome type was repeated until no additional reads were aligned between rounds of building the genome type (usually 4 rounds). At the end of the process, a single sequence was produced that represents the sample-specific genome type and contains the major nucleotide of the sample at every position of the genome. Lastly, the sequence reads were aligned to this genome type, and the alignment was used to call intrahost variants and to quantify intrahost diversity.

### Intrahost HCMV populations are diverse

**Figure 2:**
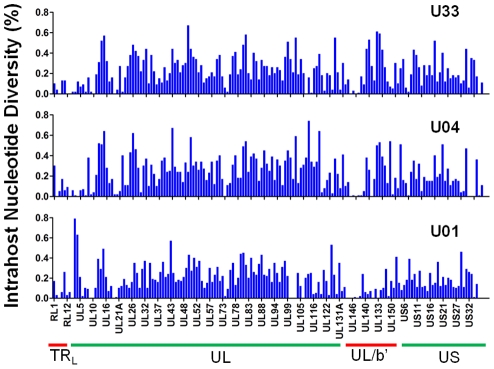
Intrahost nucleotide diversity was detected in most ORFs of the HCMV genome. Intrahost nucleotide diversity (π) was plotted for each ORF of the HCMV genome based on high throughput sequence data of clinical samples from three patients: U01, U04, and U33. The ORFs are listed in layout of the standard HCMV genome from 5′ to 3′. See Table S3 for a tabular representation of these data. Due to space constraints, not all ORFs are named on the plot. The major divisions of the HCMV genome are shown below the graph.

Intrahost variants were classified by ORF to quantify both intergenic and genome wide variability (Table 2, Table S3 and Figure 2). There were >8,500 intrahost variants in each sampled population. (Range: 8,562–13,335) (Table 2), and ∼91% of the variants were present at frequencies <0.1. We compared the levels of variants from clinical sequencing and BAC resequencing to determine the level of false positives or errors within the clinical data. The false positive rate was reduced to 6.7% with filtering (Figure S2).

**Table 2:** Intrahost diversity of hcmv populations in clinical samples: genome wide averages.

Sample	Variants^1^	π^2^ (%)	Mean Diversity (%)	π_AA_ 3 (%)	π_SYN_ 3 (%)	Variable AA Sites^4^ (%)
U01	8,562	0.18	0.17	0.14	0.04	12.3
U04	13,335	0.25	0.22	0.15	0.05	13.8
U33	10,318	0.22	0.21	0.16	0.06	14.0

1 Variants are the total intrahost single nucleotide variants.

2 π is the nucleotide diversity as calculated using the formula of Nei and Li [45].

3 π_AA_ is the intrahost amino acid diversity and π_SYN_ is the diversity of all synonymous mutations. Both were calculated in the same way as π but only using nonsynonymous or synonymous mutations, respectively.

4 Variable AA Sites are the percentage of amino acid positions in which nonsynonymous intrahost variants were detected.

Our initial analysis of the intrahost variability focused on the ORFs encoding the glycoproteins, gB (*UL55*) and gN (*UL73)*. These ORFs have well defined genotype classifications [19], [20] and previous studies have shown mixed genotype populations for these ORFs [27], [44]. Full genotypes cannot be determined using short read sequencing because linkage information is lost between regions larger than a sequence read (i.e. 72 nt in this work). We analyzed the presence and frequency of amino acid variants that are markers of gB or gN genotypes as a substitute for full-length genotype data. For example, at position 181 of gB, a lysine is unique to the gB2 genotype and an arginine is unique to gB3 [19]. K181 or R181 within gB serves as a marker of these two genotypes. The frequency of these markers is the inferred frequency of the full-length genotype. We determined that mixed genotype populations existed for the gB (*UL55*) and gN (*UL73*) loci in congenitally infected infants in agreement with previous studies [27], [44] (Table 3). However, these data represent ∼0.5% of the HCMV genome, and led us to determine whether evidence of mixed populations exists throughout the genome.

**Table 3:** Frequency of gB and gN genotype markers in high throughput sequence data.

		U01	U04	U33
**gB** 1	**gB1**	6.5%	2.9%	97.3%
	**gB2**	91.1%	93.3%	2.7%
	**gB3**	2.4%	3.8%	**-**
	**gB4**	-	-	-
**gN** 1	**gN1**	9.8%	-	3.7%
	**gN2**	2.8%	100%	2.6%
	**gN3**	5.8%	-	4.3%
	**gN4a**	78.8%	-	89.4%
	**gN4b**	-	-	-
	**gN4c**	2.8%	-	-

1 gB genotyping is based on [19], and gN genotyping is based on [20]. Unique amino acid variants for each gB or gN genotype were used as markers of the respective genotype. The frequency values of the markers in the sequenced populations are listed in the table.

**Figure 3:**
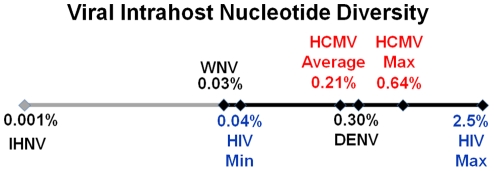
HCMV intrahost diversity is similar to RNA viruses. A logarithmic number line plotting nucleotide diversity for representative RNA viruses is shown. West Nile virus (WNV), dengue virus (DENV) and human immunodeficiency virus (HIV) were chosen because they exhibit low, mid, and high levels of π, respectively, for RNA viruses. The HIV values are from a whole genome sequencing study of 12 samples [47], and the minimum (HIV Min) and maximum (HIV Max) values are shown. HCMV Average is the genome wide average across patients for π and HCMV Max is the maximum ORF value obtained from Illumina sequencing. See Table S4 for a more thorough list of nucleotide diversity values for RNA virus populations.

To further define the intrahost diversity of HCMV populations, we first analyzed the genome wide data at the nucleotide level. We used the measures of nucleotide diversity (π) [45] and mean diversity [46], which were calculated as averages for all ORFs of the HCMV genome (Tables 2 and S3 and Figure 2). π is the average pairwise distance of sequences in the population, and mean diversity is the percentage of variant sequence within the population. The genome wide average for π for the 3 samples was 0.22% (Range: 0.18%–0.25%). As a point of comparison, this value is similar to the genome wide π for HIV [47] and the single ORF intrahost π of other RNA viruses, such as hepatitis C, dengue, and West Nile [48], [49], [50], [51] (Figure 3 and Table S4). Single ORF intrahost π was as high as 0.64% for HCMV. The HCMV genome wide mean diversity was 0.20% (Range: 0.17%–0.22) and is similar to that of HIV-1 and dengue virus [46], [50]. Figure 2 also reveals that intrahost diversity was not limited to a few loci but was found within most ORFs. The ORFs encoding gB (*UL55*) and gN (*UL73*) were in the 32^nd^ and 20^th^ percentile for ORF intrahost diversity, respectively, (Table S3) and do not reflect the genome wide diversity. Therefore, HCMV populations are variable and using unbiased, genome wide data for studying that diversity offers an advantage over previous techniques that have focused on a limited set of loci.

**Figure 4:**
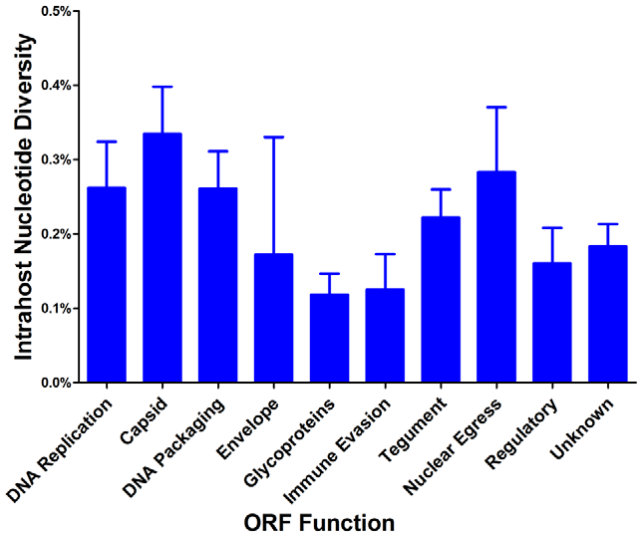
HCMV intrahost nucleotide diversity is significantly correlated with ORF function. Intrahost nucleotide diversity was calculated for each ORF of the HCMV genome. The ORFs were then grouped by function and average nucleotide diversity was calculated across all three patients. Error bars represent the 95% confidence interval for the calculated mean. 1-factor ANOVA test for significance: p <0.0001.

We grouped ORFs by gene product function or expression kinetics using the classification of Sylwester et al. [52] to further investigate the patterns of intrahost diversity (Figures 4 and S6). However, there was considerable variation of sequencing depth of some ORFs (Table S3) raising the possibility that uneven sequencing depths could influence this analysis of diversity. Indeed, there was a correlation between nucleotide diversity of an ORF and the extremes of sequencing depth (Figures S5A). To reduce the influence of excessive depth on the analysis, we focused on ORFs with sequencing depths between 15 and 1200 (n = 338) (Figure S5B). In this range, the influence of depth on nucleotide diversity will be ∼.01%, which is approximately the level of noise generated from errors in BAC resequencing. After selecting for ORFs sequenced to depths within this range, we did not observe significant difference nucleotide diversity across expression class. However, we did find a statistically significant association between ORF function and intrahost nucleotide diversity (p <.0001) (Figure 4 and S6). ORFs encoding glycoproteins showed a reduced level of intrahost nucleotide diversity. This latter result was unexpected given that glycoproteins were the most frequently analyzed in earlier studies of intrahost variability.

**Figure 5:**
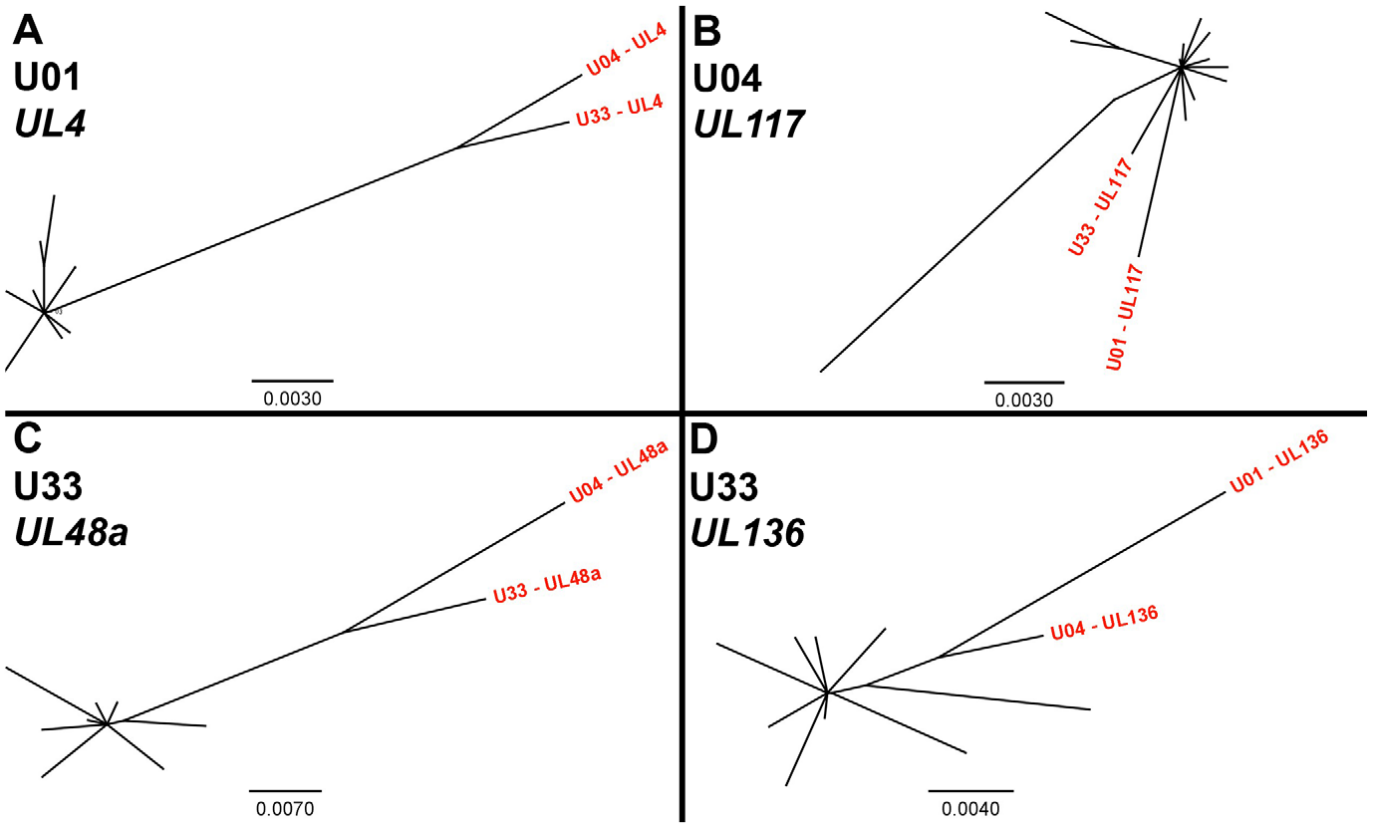
Unrooted phylogenetic trees of HCMV populations show varying levels of diversity. Highly variable ORFs in the high throughput sequencing dataset of each patient sample were selected for clonal Sanger sequencing. Unrooted phylogenetic trees were generated from the data, and major genotype sequence data from the other patient samples in this study were included to provide perspective (shown in red text). The trees from some datasets showed clones that were unique but closely related (**5A, 5C**). The clones from others datasets were more divergent, such that divergence within a patient sample was larger than divergence between patient samples (**5B, 5D**). Examples of both patterns could be seen at different ORFs from the same patient sample (**5C, 5D**). Phylogentic trees generated from all Sanger datasets can be seen in Figure S7. Units for scale bars are substitutions per site.

To confirm the results obtained via high throughput sequencing, we assayed for π and genotype distribution by clonal Sanger sequencing of three highly variable ORFs in each patient sample. We found that the major genotype detected in both methods is the same (data not shown). Also, the values for π determined by both high throughput and Sanger sequencing were generally similar for each ORF (Table 4). Clonal Sanger sequencing of these ORFs revealed a high density of unique genotypes in the clinical samples, with as many as 13 unique genotypes from 20 clones. The Sanger sequence data was also used to generate unrooted phylogenetic trees (Figures 5 and S7). Within the trees, we have included major genotype sequence data from the other patient samples in this study to provide perspective on the diversity of the clones. In some Sanger datasets, the diversity of clones could be explained by one or two mutational steps from the major genotype (Figure 5A). Other datasets revealed clones *within* a patient sample that were more divergent than sequences *among* patient samples (Figure 5B). This result could represent a highly mutagenic viral population, a co-infection with two or more strains, mixtures of viral variants from different compartments, or a combination of these mechanisms. An interesting side note is that, in a single patient sample, there is evidence for diversity from a few mutational events (Figure 5C), and possible evidence of co-infections (Figure 5D). Thus, the mechanism(s) that leads to the diversity of HCMV populations may be complex.

**Table 4:** Intrahost nucleotide diversity as measured by two sequencing methods.

Patient Sample	ORF	π (Illumina)^1^	π (Sanger)^2^	Clones Sequenced^3^	Unique Genotypes^3^
**U01**	***UL2***	0.59%	0.50%	20	10
	***UL4***	0.53%	0.51%	19	7
	***UL51***	0.40%	0.33%	20	9
**U04**	***UL117***	0.64%	0.40%	19	9
	***UL15a***	0.64%	0.58%	19	8
	***UL26***	0.62%	0.52%	20	9
**U33**	***UL48a***	0.62%	0.52%	19	9
	***UL136***	0.61%	0.48%	18	9
	***UL15a***	0.57%	0.57%	20	13

1 π (Illumina) is the nucleotide diversity calculated from Illumina high throughput sequence data.

2 π (Sanger) is the nucleotide diversity calculated from clonal Sanger sequencing.

3 The number of clones sequenced and unique genotypes refers to the Sanger sequencing data.

Because the coding sequence of HCMV populations appeared to be highly variable, we next investigated whether there were differences in variability between coding and non-coding regions of the genome. For this analysis, coding regions were defined as protein coding sequences, and non-coding regions comprised the remainder of the genome. Thus, the non-coding regions likely contain functionally important sequences due to the inclusion of regions such as the origin of replication, transcription factor binding sites and miRNA sequences. Using these parameters, we found that there was a statistically significant difference between intrahost diversity of the coding and non-coding regions (Table 5). The coding regions had higher nucleotide and mean diversity values than the non-coding regions; however, the average frequency of coding variants was significantly less than the average frequency of non-coding variants. Although the differences in values for these summary statistics are small, as seen in the U04 population, it should be noted that coding and non-coding variants are interspersed across the genome. Thus, this proximity should allow for statistical robustness and may reflect a fine-scale mechanism regulating the amount and frequency of coding and non-coding variants.

**Table 5:** Distribution of intrahost variants in coding and non-coding regions of the HCMV genome.

U01	Non-coding	Variant Sites	Variants	Length (bp)	π	Mean Diversity	Average Variant Frequency	p value (Mean Diversity)^1^	p value (Frequency)^2^
		1222	69544	48552	0.14%	0.12%	6.44%		
	**Coding**	**Variant Sites**	**Variants**	**Length (bp)**	**π**	**Mean Diversity**	**Average Variant Frequency**	<.0001	<.0001
		7462	498257	187075	0.18%	0.19%	5.02%		
**U04**	**Non-coding**	**Variant Sites**	**Variants**	**Length (bp)**	**π**	**Mean Diversity**	**Average Variant Frequency**	**p value (Mean Diversity)** 1	**p value (Frequency)** 2
		2430	115228	48521	0.24%	0.25%	5.44%		
	**Coding**	**Variant Sites**	**Variants**	**Length (bp)**	**π**	**Mean Diversity**	**Average Variant Frequency**	<.0001	<.0001
		11152	797847	187117	0.25%	0.26%	4.50%		
**U33**	**Non-coding**	**Variant Sites**	**Variants**	**Length (bp)**	**π**	**Mean Diversity**	**Average Variant Frequency**	**p value (Mean Diversity)** 1	**p value (Frequency)** 2
		1474	51079	48451	0.17%	0.13%	7.07%		
	**Coding**	**Variant Sites**	**Variants**	**Length (bp)**	**π**	**Mean Diversity**	**Average Variant Frequency**	<.0001	<.0001
		9043	904298	186841	0.22%	0.27%	5.23%		

1 p value for a Z-test of proportions of the mean diversity of the non-coding and coding variants.

2 p value for a two-tailed Mann-Whitney test for the distribution of variant frequencies of the

We next investigated the clinical HCMV populations at the amino acid level. The average intrahost amino acid diversity (π_AA_) was 0.18% (Table 2), which is comparable to RNA viruses such as dengue and West Nile [48], [50]. The diversity at nonsynonymous sites (π_AA_) was ∼3-fold higher than at synonymous sites (π_SYN_), suggestive of a slight excess of nonsynonymous mutations within the HCMV populations. The genome wide average for the percentage of amino acid sites that exhibited intrahost variability was 13.4% (Range: 12.3%–14.0%) (Table 2). This value reveals the substantial variation in intrahost coding potential of HCMV populations. Taken together, these data support a model of HCMV existing as diverse populations at both the nucleotide and amino acid levels. This result is novel for a large dsDNA virus, which encodes a DNA polymerase with exonuclease activity [53].

### Evidence of positive selection in HCMV intrahost populations

**Figure 6:**
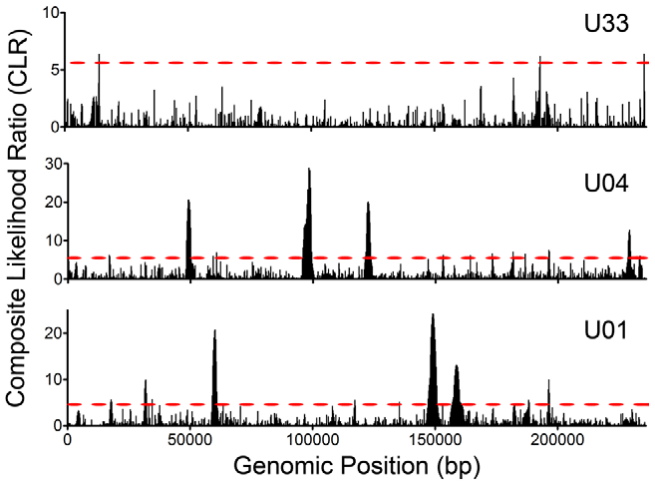
Selective Sweeps were detected within HCMV intrahost populations. Intrahost variant data was processed with the model of Nielsen *et al* and composite likelihood ratios (CLRs) were calculated for 235 bp windows across the HCMV genome. CLRs are measures of the probability of a selective sweep within a genomic region. Simulations were run to determine the threshold values for significance and these values are depicted as red, dashed lines across the graphs (see Figure S8 for a presentation of the simulation data). Each CLR above the threshold is considered significant and is indicative of a selective sweep occurring within the window. See Table S5 for a tabular presentation of ORFs located in statistically significant regions and the corresponding CLR and p values.

Having found significant levels of intrahost variability, we felt it was important to determine whether the patterns in variability were the result of genetic drift (i.e. neutrality) or if selection could explain the observed variant frequency patterns in the populations. We applied the model of Nielsen *et al* [54] to detect selective sweeps within the genome wide variant data. Selective sweeps are caused by positive selection and result in reduced variability around the region under selection [55], [56]. Importantly, the test of Nielsen *et al* is robust to demographic effects. This is a critical function because the HCMV populations under study have most likely undergone significant recent demographic changes, such as population bottlenecks and expansions associated with primary infection. The Nielsen approach is an outlier test that calculates the likelihood of a selective sweep based on the distribution of variant frequencies within a region as compared to the genome as a whole. The composite likelihood ratio (CLR) of the region is a measure of this comparison, with higher CLR values indicating the region is a more extreme outlier and thus, more likely a target of positive selection. Applying the model of Nielsen *et al* to the HCMV genome wide data, we identified an average of 9 ORFs per population (Range: 2–15) under statistically significant positive selection (Figures 6 and S8 and Table S5), including *UL83* (pp65) and *UL123* (IE1). While there was no overlap between the positive selected ORFs in the three samples, there was evidence of overlap in protein function. For example, *UL102* in the U01 sample and *UL105* in the U04 sample were targets of selective sweeps, and protein products of both ORFs are subunits of the helicase-primase complex. Many of the ORFs highlighted in this analysis have either poorly defined or no known function.

### Interhost HCMV variability and selection

The generation of HCMV sequence data from urine specimens allowed for genome wide analysis of *interhost* polymorphisms across clinical samples, as opposed to those observed in laboratory passaged strains. For this analysis, polymorphisms were defined as variants from the HCMV reference sequence with frequencies >0.5, and are the same class of variants previously incorporated into a sample specific genome type. By resequencing HCMV BACs, we determined that the error rate for calling polymorphisms is 0.028%., i.e., ∼65 erroneous polymorphisms are called within a 236,000 bp genome type (Table S2). On average, there were ∼2600 polymorphisms per genome type resulting in an interhost variability of 1.1% at either the nucleotide or amino acid level (Table 6). Only 7.9% (612 of 7,780) of the nucleotide polymorphisms and 1.2% (25 of 2,129) of the amino acid polymorphisms were common among the 3 samples. This result shows that most of the polymorphisms are not only different between clinical populations and a laboratory passaged strain (Merlin), but they appear to be uniquely associated with the specific environments of the viral populations. Thus, these findings are consistent with previous work showing diversity of the HCMV species [5].

**Table 6:** Interhost variability of hcmv populations in clinical samples: genome wide averages.

Patient	Polymorphisms	π^1^	dN/dS^2^	π_AA_ 3
U01	2,909	1.20%	0.10	1.05%
U04	2,347	0.97%	0.14	1.09%
U33	2,524	1.01%	0.14	1.23%

1 π is the nucleotide diversity between the consensus sequence from each clinical HCMV population and the HCMV reference sequence (Merlin) as calculated using the formula of Nei and Li [45].

2 dN/dS was calculated using the formula of Nei and Gojobori [73].

3 π_AA_ is the interhost amino acid diversity between clinical major genome type and the HCMV reference sequence (Merlin). It is calculated with the same formula as π but only nonsynonymous mutations are included.

**Figure 7:**
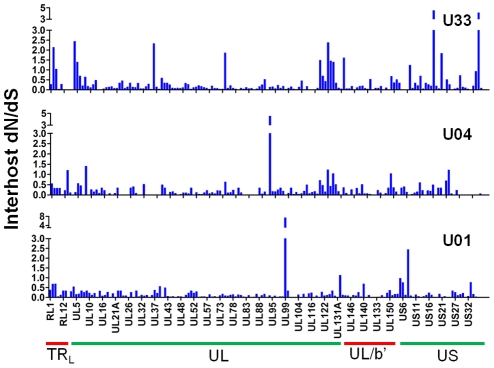
A majority of ORFs appeared to be under negative selection based on interhost dN/dS values. Interhost dN/dS were plotted for each ORF of the HCMV genome based on high throughput sequence data of clinical samples from three patients: U01, U04, and U33. The ORFs are listed in layout of the standard HCMV genome from left to right. See Table S6 for a tabular representation of these data. Due to space constraints, not all ORFs are named on the plot. The major divisions of the HCMV genome are shown below the graph.

**Figure 8:**
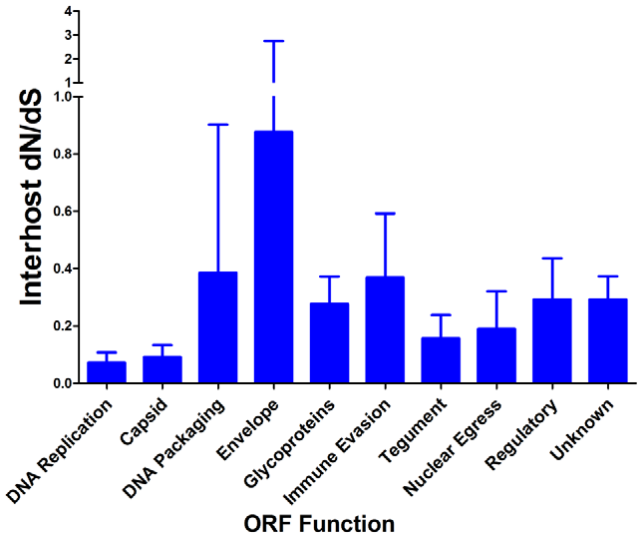
HCMV interhost dN/dS is significantly correlated with ORF function. Interhost dN/dS values were calculated for each ORF of the HCMV genome. The ORFs were then grouped by function and average interhost dN/dS was calculated across all three patients. Error bars represent the 95% confidence interval for the calculated mean. 1-factor ANOVA test for significance: p = 0.0002.

Next, we wanted to determine whether there is evidence of selection within the interhost sequence data. Previously, single ORFs of the HCMV genome have exhibited dN/dS ratios of less than 1 [57], [58], suggestive of negative selection. Using the genomic data, we calculated dN/dS values for all ORFs of the HCMV genome and also calculated a genome wide average. In agreement with previous studies [57], [58] **,** the genome wide average dN/dS values were significantly below 1 (p <0.0001, G-test) (Table 6, Table S6 and Figure 7). Approximately 5% of ORFs exhibited dN/dS values greater than 1, which is suggestive of positive selection. To find patterns in the genome wide dN/dS values, ORFs were classified according to protein product function and expression kinetics (Figures 8 and S9). No significant association was seen between dN/dS and expression kinetics, but a highly significant association was observed between protein product function and dN/dS (p = 0.0002). Envelope proteins exhibited elevated dN/dS values and DNA replication proteins showed low dN/dS values (Figure 8).

We next used the McDonald-Kreitman (MK) test on the clinical sequence data to further analyze selective pressures. The input data for the MK test are the divergent (i.e. *interspecies*) nonsynonymous (D_N_) and synonymous (D_S_) mutations and the polymorphic (i.e. *intraspecies*) nonsynonymous (P_N_) and synonymous (P_S_) mutations [59]. Due to the inclusion of both polymorphic and divergent mutations, the MK test is a more sensitive test for selection than the dN/dS statistic. A 2x2 contingency table of the values is used to calculate significance of the mutational pattern and the respective ratios provide information regarding the direction of the test rejection. For example, positive selection is generally regarded to result in a (D_N_/D_S_)/(P_N_/P_S_) ratio >1, while negative selection results in a ratio <1.

A genome wide MK test was performed using sequences of all orthologous ORFs (n = 160) from Merlin and the three clinical samples with the inclusion of chimpanzee cytomegalovirus (CCMV) as the outgroup. Approximately 65% (n = 104) of ORFs were scored as neutral in this test. ORFs yielding (D_N_/D_S_)/(P_N_/P_S_) ratios significantly >1 were ∼4-fold more frequent than ORFs producing ratios significantly <1 (n = 45 and n = 11, respectively) (Table S7). This pattern could result from positive selection. However, considering the statistically robust, non-neutral dN/dS values, there is also widespread evidence of pervasive negative selection. Taken together, the results suggest that positive selection has driven the fixation of HCMV-specific mutations, and contributed to the divergence of the HCMV and CCMV species. However, demographic effects could also contribute to the observed mutational patterns and cannot be completely ruled out from these analyses, though considering inter-digitated synonymous and nonsynonymous sites ought to allow for a robust statistic.

## Discussion

High throughput sequencing has dramatically increased the number of genomes sequenced and is a useful tool for analyzing populations present within various environments. Our work represents the first use of high throughput sequencing technology to study the intrahost genomic populations of a large DNA virus in clinical samples. We observed substantial intrahost variability that was found throughout the HCMV genome and found evidence of selection both at the intrahost and interhost levels.

An unexpected finding of this study was that almost every ORF of the HCMV genome showed some level of intrahost diversity in the three populations that were sampled. Thus, these results are an important extension of previous work that has revealed intrahost diversity within a small number of ORFs, including gB and gN [27], [44]. However, the present data suggest that genotyping may not be a reliable surrogate for measures of HCMV diversity in clinical specimens. For example, the gB and gN genotype data in Table 3 suggest that sample U01 is genetically the most diverse and U04 is the least diverse. However, Table 2 shows the opposite to be true. U01 is the least diverse and U04 is the most diverse for HCMV on genome wide scales.

By quantitating variability using the measure of nucleotide diversity, it can be seen how the intrahost diversity of HCMV is comparable to those of RNA viruses, including HIV. The similarity in values is striking considering the common assumption that RNA viruses exist in more highly diverse populations than DNA viruses due to the lower replication fidelity of RNA genomes. Thus, this work leads to a questioning of the source of the diversity observed in HCMV populations. One possibility is the prevalence of high mutation rates during replication of viral DNA genomes, similar to RNA viruses. This possibility does not seem likely considering that HCMV encodes a DNA polymerase with proofreading activity [53]. A second possibility is low mutation rates but high levels of replication, leading to an accumulation of mutations. In support of this model, it is suspected that only a single or very few virions cross the placenta to initiate a congenital infection. At the time of collection (<2 weeks postnatally), the samples contained ∼10^7^ HCMV genome copies per mL of urine (data not shown). Thus, there had been many rounds of recent replication within the new host before the populations were sampled, which could lead to the accumulation of many variants even with a low mutation rate. Alternatively, the diversity could result from re-infection or co-infection. The phylogenetic trees of select ORFs (Figure 5 and S7) suggest that some ORFs are highly divergent from a central population of genotypes, which suggests re/co-infection events. However, phylogenetic trees for other ORFs reveal highly similar clones. More experiments are needed to sort out these possibilities.

Although the source of diversity is currently unclear, the existence of high intrahost diversity does lead to models of HCMV evolution. Creation of *de novo* mutations is stochastic and most likely occurs rarely, as suggested by the proofreading DNA polymerase encoded by HCMV. A high level of standing or pre-existing variation means that a pool of variants exists prior to the introduction of a new selective pressure. A low frequency variant(s) could quickly rise to high frequency because the selection coefficient of this allele could be increased under the new environmental conditions. Thus, diversity should offer a rapid mechanism of evolution for the virus in an environment of changing selective pressures. Alternatively, the low frequency variants could simply represent non-functional genomes or be reduced in frequency by negative selection. Data showing that the frequency of variants in coding regions is significantly lower than the frequency of variants in non-coding regions of the viral genome (Table 5) are consistent with this explanation. Again, it is possible that changing selective pressures could reverse this effect and cause a change in frequency of these variants. Future experiments should test the effect of changing selective pressures on the frequency of pre-existing variants in the population.

Analysis of the sequence data revealed evidence of selection within the viral populations. The results of the selective sweep analysis (Figure 6 and Table S5) are intriguing in the context of host-pathogen dynamics. Both *UL123*, encoding IE1, and *UL83,* encoding pp65, were found to be within regions of selective sweeps in one patient sample (U04). These proteins are demonstrated targets of CD8^+^ T cells in neonates with congenital infection [60] and suggest an immune-mediated mechanism of selection. This is the first evidence that known HCMV immune targets are also targets of positive selection. The selective sweep analysis also detected many ORFs with no known function. Whether these ORFs are under immune selection or are targets of positive selection for other reasons, such as tropism or viral replication, is still unknown.

We found evidence of both positive and negative selection within the genome when comparing interhost variation. The results suggest a model in which positive selection contributed to the divergence across the HCMV species, but genetic stability of the viral species is maintained with negative selection. Contrasting these long term selective forces to the observed high level of standing variation of the intrahost populations may lead to a clearer interpretation of the results. As mentioned above, the standing variation potentially reduces the time of adaptation to a novel environment or pressure. However, the negative selection acting on the variants may balance this phenomenon and prevent deleterious mutations from reducing the fitness of the overall HCMV species.

Two groups have recently reported using high throughput sequencing to study HCMV from clinical material. In the report by Cunningham *et al* [61], a major genome type sequence was generated from clinical material. In contrast, Gorzer *et al* [44]. studied genetic populations at three loci. These approaches are complementary to that presented here in which we sequenced HCMV populations on genome wide scales. As compared to the work of Cunningham *et al*, our study requires PCR amplification to select for HCMV DNA, which produces more HCMV-specific sequence data on a single sequencing run and greater depth of the viral population. This increased sequencing depth allows for a more accurate detection of minor variants within the population (Table S1). However, the approach by Cunningham *et al* differs from ours in that it allows for a more rapid sequencing of the major genome type, thereby producing greater sequence information about the HCMV species. In contrast, Gorzer *et al* sequenced three loci of the HCMV genome to a greater depth than our study, leading to higher levels of confidence in detecting minor and rare variants. However, our use of a genome-wide approach allows for unbiased detection of variability. As proof of the power of this approach, a commonly studied variable ORF, such as *UL73* (gN), is in the lowest quintile for intrahost diversity, while many of the ORFs with the highest intrahost diversity have not been studied for variability. Therefore, a genome-wide study can highlight loci for future studies using ultra-deep sequencing.

The results presented here suggest that diversity of DNA virus populations should be studied more thoroughly to determine the universality of the high level of variability. For example, in this study we sampled HCMV populations from urine of congenitally infected children. It is unknown if the genomic populations sampled from urine are representative of the populations in other compartments of the host. Also, the levels of replication during congenital infections are very high, such that the diversity observed in asymptomatic, adult hosts may be much lower due to lower levels of replication and, therefore, fewer opportunities for mutagenesis. Alternatively, the chance of co- or re-infection in adults is much higher, possibly leading to more diverse populations. Others have shown that Marek's disease, another herpesvirus, virus exists as a collection of mixed genotypes in culture [62]. Thus, there is evidence of a similar phenomenon. Whether high diversity, mixed genotype populations exist for other herpesviruses or other dsDNA viruses outside of this family remains to be seen.

## Materials and Methods

### Ethics statement

Clinical specimens were obtained from neonates with congenital HCMV infection and de-identified prior to receipt by the investigators. Specimens were gathered as part of a standard clinical procedure. None of the investigators were involved in specimen collection. The use of these specimens for research was approved by the University of Massachusetts Medical School Institutional Review Board (IRB Docket # 10778).

### Patient population, collection of samples and cloned viral DNA

Neonates within two weeks of age were diagnosed with congenital HCMV infection at the request of their respective care providers. The University of Massachusetts Memorial Health Center clinical virology laboratory performed diagnostic virus isolation. De-identified urine samples were then used for this study. No clinical information about the infants was available. Samples were stored at −80°C until DNA purification. DNA was purified using a Qiagen Blood and Tissue Kit using the standard protocol. HCMV BAC DNA has been described previously [40] and was kindly provided by Tom Shenk (Princeton University). Isolation of BAC DNA from *E. coli* strains was performed as described [63].

### Amplification of HCMV DNA

We constructed a set of primer pairs spanning the entire HCMV genome. Primers were designed to anneal to conserved sites of the HCMV genomes, based on publicly available HCMV sequences. These databases included the sequence of an HCMV genome type (Strain 3157) that was produced directly from clinical material without amplification [61]. Primer homology with this strain supports the assertion that the chosen sites are found in wild type strains, and will reduce primer mismatch bias. Amplicons overlapped by ∼100–500 bp such that sequence was generated at primer binding sites from the adjoining amplicon. Using this overlap data, primers were reevaluated and redesigned primers as necessary, given that these new data potentially represent thousands of unique HCMV genomes per experiment. Lastly, primers were designed to have no or low homology to both human sequence and any other possible contaminating DNA sources, such as other herpesviruses or common human parasites and commensal bacteria.

Most amplicons were ∼6 kilobases (kb). Some were reduced to 3 kb if the original longer amplicon either gave no/weak amplification or non-specific products as determined by Sanger sequencing. Primer sequences used in this study are listed in Table S8. For BAC and clinical sample PCR amplification, initial PCR reactions were carried out using serially diluted templates to determine the lowest quantity necessary for efficient amplification. Quantitative PCR was performed using primers and probes described previously [64] and it was determined that each reaction contained ∼1300 HCMV genomes. The conditions for PCR were as follows: 1X PfuUltra II PCR buffer, 0.25 mM each dNTP (NEB), .25 uM each primer (IDT DNA), 0.5 uL PfuUltra II Polymerase (Agilent) and 1 M betaine. A touchdown PCR was run on an Eppendorf Mastercycler ep gradient S with the following program for all reactions: 98°C for 2 min, 5 cycles of 98°C for 30 s, 63°C (decreasing by 1°/cycle) for 30 s, 72°C for 2 min, followed by 25 cycles of 98°C for 30 s, 58°C for 30 s and 72°C for 2 min, with a 10 min final extension at 72°C. All amplified products were size-selected on agarose gels and gel purified. Because insertions or deletions could produce amplicons of visibly different sizes than expected, we used direct Sanger sequencing of questionable amplicons to test for presence of the expected HCMV sequence. After amplification of the HCMV genome, all amplicons were quantified on a Nanodrop 1000, pooled in equimolar proportions and used as substrate in Illumina sequencing.

### Illumina sequencing

The DNA in pooled amplicons was sheared by sonication on a Sonic Dismembrator 550 (Fisher) until the median size was ∼350 bp. The DNA library was prepared as stated previously [65]. Briefly, DNA was end-repaired using the End-Repair Enzyme Mix (NEB), and A-tailed using the ATP and Klenow (exo^-^) (NEB). Adapters with appropriate barcodes were ligated onto the modified DNA ends. The library was then size selected on a 2% agarose gel, to produce a library with a median size of 350 bp+/−50 bp. The library was amplified with Illumina primers (P/N 1003454) (www.illumina.com). Once prepared, the libraries were combined in appropriate ratios and submitted for paired-end sequencing on the Illumina GAII. A Toledo strain amplicon set was included as an internal control for measuring error rates.

### BAC resequencing and development of methodology

HCMV BAC DNAs of the AD169 and Toledo strains were PCR amplified and processed for sequencing as described above. The barcoded DNAs were then sequenced on a single lane of the Illumina GAII. Output sequences from the Illumina GAII were first converted from Illumina FASTQ format to Sanger standard FASTQ and were then separated based on barcode sequences, which were subsequently trimmed before subsequent processing. The sequences were then aligned to either the AD169 BAC (GenBank # AC146999) or Toledo BAC (GenBank # AC146905) using Novoalign (Novocraft). The alignment data were then ported to MAQ through the Novo2MAQ utility (Novocraft) and downstream analyses were performed with the MAQ software suite [66]. The pileup output from the alignment was then analyzed to call any mismatches between the sequence reads and the reference genome. All mismatches from this output have an associated basecall quality, mapping quality, local depth, number of mismatch occurrences and mismatch frequency. The basecall quality and mapping quality are calculated by the sequencing and alignment software, respectively.

### Development of variant filtering algorithm

We used HCMV-BACs as templates for PCR amplification and paired-end sequencing on the Illumina GAII to develop an algorithm that would reduce error. The output was 108 megabases of HCMV sequence or the equivalent of approximately 466 HCMV genomes (Table 1). The data were aligned to the appropriate reference genome using Novoalign and MAQ. Using these data, we developed a variant filtering algorithm. This algorithm has been designed to filter the mismatch output from the alignment stage and aid in sorting “true” variants in the viral population from those mismatches created by PCR or sequencing errors. We produced *in silico* models of mixed viral populations in which the AD169:Toledo ratio was 1:1, 1:10, 1:100, 1:200, and 1:1000. Thresholds for minimum basecall quality (≥30), mapping quality (≥89), depth (≥15), mismatch count (≥3) and mismtach frequency (≥0.019) were found to minimize false positives. With these conservative thresholds, we had a detection rate of up to 75%, suggesting that the variants detected in clinical samples will under-represent the true level of variation in the populations. However, the number of false positives was very low in these *in silico* experiments even when the input minor genome was 1% of the population (Table S1). Modeling of two genotype mixed populations, like those represented in Table S1, illustrates a worst case scenario for a false positive rate. In Table S1, there are two types of variants: “true” variants, sourced from the minor genome type, and errors resulting from PCR or sequencing. The absolute level of true variants will be dependent on the number of minor genome types; as the number of minor genome types increases, the number of true variants also increases. The number of errors, though, is a function of PCR and sequencing and should be independent of the number of minor genome types. Thus, the ratio of errors to true variants (the false positive rate) will decrease as the number of minor genome types increases. In this modeling experiment, there is only one minor genome type and thus, we are recording the upper limit of false positive rates of a mixed genome type population. From the Sanger dataset (Table 4), it was shown that the populations studied are comprised of many genotypes (e.g. 13 unique genotypes from 20 clones), not just one minor genotype. Thus, this modeling experiment overestimates the actual false positive rate of the clinical data.

It was possible that the relatively high G:C content of the HCMV genome could alter error rates across the genome, and should be addressed by the error filtering protocol. However, we did not detect a relationship between error rates and G:C content from the BAC resequencing data (data not shown). We did observe an association between G:C content and depth, with reduced depth at very low (20%) or very high (>80%) G:C content (data not shown). This characteristic of the Illumina platform has been documented previously [67]. We corrected for differences in depth when analyzing the intrahost populations (Figure S5) so that changes in depth associated with G:C content should not alter our analyses.

### Performance of quantitative high throughput sequencing

To determine the quantitative capabilities of our methodology, we combined Toledo and AD169 BAC DNA in ratios of 1:10 and 1:100 as templates for PCR amplification (with Toledo present as the major genome) and then amplified two regions of the genome using our PCR amplification technique. These two regions represent ∼6 kb of the HCMV genome and have a GC content of 58%, approximately equal to the genome wide average of 57%. In these regions, there are 118 sites of mismatch between the Toledo and AD169 genomes. The amplification products were processed and sequenced using the Illumina GAII platform and the output was aligned to the Toledo genome. We ran the data through our variant filtering algorithm to detect the minor variants in the sequence population (i.e. AD169-derived sequence). Our data revealed a 48% detection rate when the minor genome is present as 10% of the PCR template and a 38% detection rate when present as 1% (Table S9). The relatively low detection rate is a consequence of the stringency of the filtering algorithm we developed. The frequency of the minor variants detected in the output sequence was approximately equal to their frequency in the input DNA. These data show that this methodology is suitable for detection and quantitative description of variants in populations.

### Calling sample specific genome type of clinical samples

A schematic for calling genome types is shown in Figure S4. The high throughput sequencing reads were initially aligned to Merlin (Ref Seq ID: NC_006273). Output from this initial alignment was used to call variants with frequency >0.5 at every position of the genome because these variants were interpreted to best represent the major allele of the sample. Sites that did not have an allele with a frequency >0.5 were left as uncalled bases (N), and were excluded from intrahost diversity measurements since they represent tri- or quad-allelic sites. The high frequency variants were incorporated into a sample specific genome type. Reads that did not initially align were used as substrate for *de novo* contiguous sequence (contigs) assembly using SHARCGS [68]. These contigs were then aligned to the sample specific genome type using Geneious [69] and incorporated into the genome if sequence identity was found. Using this strategy, we were able to remove up to ∼1 kb of uncalled bases from the genome type. The sample specific genome type was used in another round of alignment of the sample's sequencing reads. With this strategy, we observed a 1–6% increase in the number of aligned reads after this round as compared to the initial alignment to Merlin. Because more reads aligned, additional high frequency variants were called. The high frequency variants were incorporated into the sample specific genome type and again contigs were aligned to the genome type. This process was repeated until no additional reads aligned between rounds of building the sequence (usually 4 rounds were required). At the end of the process, a single specific genome type was created for each sample, which incorporates all high frequency variants found within. It is unknown if the sample specific genome type represents any single genome within the sample because linkage information is lost from short read sequencing. The sample specific genome type is a computational tool that aids in the alignment of short reads, particularly when a pre-existing reference sequence is unavailable or is divergent from the sample.

### Analysis of false positive rate of clinical samples

Variants were called from the clinical sequencing data or BAC resequencing data through filtering with the variant caller algorithm. All alignments used to generate the data were normalized to an average depth of 200 genome equivalents. A depth of 200 was chosen because the lowest depth of an included dataset was ∼200 (i.e., AD169 BAC resequencing), so the ceiling was set to normalize across datasets. Mismatches from BAC resequencing were assumed to be errors and mismatches from clinical sequencing were assumed to be either errors or true variants. Without filtering, BAC resequencing generated, on average, 106,485 called mismatches and clinical sequencing generated 116,594 mismatches (Figures S2A, S2C). Therefore, we estimate a false positive rate in the unfiltered clinical data of 91.3% (106,485 of 116,594). However, filtering with the variant caller reduced estimated false positives to 6.7% of the clinical variants within populations (Figures S2B, S2D).

To determine the error rates of calling interhost polymorphisms (frequency >0.5), a similar analysis of the BAC resequencing data was undertaken. We determined that the error rate for calling polymorphisms is 0.028%, or ∼65 erroneous polymorphisms per genome (Table S2). On average, interhost HCMV sequence data contained >2300 polymorphisms per genome.

It should be noted that the error rate for calling interhost polymorphisms is significantly lower than the error rate for calling intrahost variants (0.028% vs. 6.7%). Intrahost variants must occur at least 3 times as part of the filtering strategy. However, interhost polymorphisms, because they are present at frequency >0.5 and the minimum depth is 15, must occur more than 8 times to be called. Because random errors generated by PCR or sequencing will most likely be rare, the possibility of random errors occurring ≥8 times and occurring in >50% of reads is low. Thus, a lower percentage of errors are included in the interhost polymorphism data then the intrahost variant data.

### Measurement of positive selection in intrahost populations

The genome wide intrahost variant data was analyzed using the program SweepFinder (http://people.binf.ku.dk/rasmus/webpage/sf.html), which implements the methods of Nielsen *et al*. [54] and outputs the position, selection coefficient and composite likelihood ratios (CLRs) of genomic regions. CLRs are measures of the probability of a selective sweep within a genomic region. To determine the significance of the data, 1000 simulations were performed under a standard neutral model using the ms program [70]. A set of simulations was run for each clinical sample population, in which the number of segregating sites and value of θ (Watterson estimator) of the simulation equaled the corresponding values calculated from the clinical samples. The simulation was then processed with SweepFinder, and output from this analysis (Figure S8) was used to determine p values by comparing the clinical value to the simulated outputs.

### Clonal Sanger sequencing

ORFs were chosen for clonal Sanger sequence by selecting candidate ORFs from each patient sample that displayed high intrahost variability. All clonally sequenced regions were between 500–700 bp, such that variability data could be generated in a single Sanger sequencing reaction. The regions were amplified with the appropriate primers using the PCR protocol described above, A-tailed with Kleno exo^-^ and dATP (NEB), and cloned into the Strataclone cloning vector (Stratagene). For each ORF, 20 clones were selected at random and sequenced. As a control, a 500 bp region of Toledo-BAC was amplified and clonally sequenced in the same manner. These data were then analyzed in DnaSP [71] to determine nucleotide diversity (π) and genotype distribution.

### Statistical analyses

For analysis of the association of ORF function or kinetics with intrahost nucleotide diversity or interhost dN/dS, a 1-factor ANOVA analysis was performed. A Bonferroni correction for multiple testing was carried out, where a significant p-value was considered <.05/K where K is the number of tests run per dataset. A G-test was performed on the interhost dN/dS values with the null hypothesis set as dN/dS = 1. The McDonald-Kreitman test was done using the web portal as described in [72], which performs the analysis using a Jukes-Cantor correction for divergence and the statistical analysis based on a 2×2 contingency table. A neutral model was rejected if p<0.05. A Z-test was used to determine the significance of the proportions of the mean diversity of non-coding and coding variants. The distribution of variant frequencies was analyzed by a two-tailed Mann-Whitney test.

### Availability of data

Raw sequencing reads from Illumina sequencing are deposited in the Sequence Read Archive (http://www.ncbi.nlm.nih.gov/Traces/sra/sra.cgi). Major genome types generated from this study are deposited in Genbank (http://www.ncbi.nlm.nih.gov/genbank/index.html).

## Supporting Information

Figure S1 Coverage of HCMV genome in previous sequencing studies. The HCMV genome is depicted as a grey bar, with the subdivisions of the genome shown above as black bars. The coverage of the genome from previous sequencing studies is depicted with blue bars, with each blue bar representing a sequence study and the width of the bar being proportional to the length of the sequenced region. Although some regions have been sequenced in multiple studies (for example, *UL55* (gB)), for the purposes of this figure, we show the data from the study that sequenced the largest region. The data used to construct this figure are from [1], [2], [3], [4], [5], [6], [7], [8], [9], [10], [11], [12], [13], [14], [15], [16], which are listed in Text S1.
(0.04 MB TIF)

Figure S2 Single nucleotide variant counts and frequencies as a result of filtering. Variants were called from BAC resequencing or clinical sequencing alignments normalized to an average depth of 250. A. Variants of all frequencies called from the two datasets without filtering. B. Variants of all frequencies called from the two datasets after filtering with the variant caller algorithm. C. Same as A except that only variant frequencies from 0–0.2 are displayed. D. Same as B except that only variant frequencies from 0–0.2 are displayed.
(0.69 MB TIF)

Figure S3 Coverage map of HCMV genomes sequenced directly from urine samples. HCMV was sequenced from urine samples U01, U04 and U33 and the predicted coverage of the genomes was calculated. The black bars show a one dimensional representation of the genome, while the blue curve above is indicative of both the coverage and quality of sequence data across the genome. The presence of a blue curve indicates coverage of a base and the height of the blue curve is proportional to the quality. The major divisions of the HCMV genome are shown below the graph.
(0.15 MB TIF)

Figure S4 Flowchart of genome type calling and detection of intrahost variants. Alignment of the high throughput sequence data begins by using the HCMV reference sequence (Merlin, Ref Seq ID: NC_006273). Mismatches between the reference sequence and high throughput sequence reads are identified from the alignment and data about all mismatches (depicted as C or T in the figure) are outputted into a pileup file. The pileup files is processed with a variant filter protocol that uses threshold values for basecall quality, mapping quality, depth, mismatch frequency, and the number of mismatch occurrences. Mismatches with characteristics above these threshold values are outputted by the variant filter. Mismatches from this filtering are either high frequency (frequency >0.5, Red C) or low frequency (frequency <0.5, Black T). The high frequency mismatches are interpreted to be sample specific polymorphisms, and are incorporated into the sample specific genome type. Additionally, unaligned reads are used to build contiguous sequences (contigs) and are incorporated into the sample specific genome type if showing homology to the sample specific genome type. The sample specific genome type is then used as the reference sequence for additional rounds of alignment of the sequence reads. Again, high frequency polymorphisms are incorporated into the genome type, and contigs are built and assembled onto the genome type. This process is repeated until no additional high throughput sequence reads align to the genome type. The genome type is exported to create the final sample specific genome type (Blue line with incorporated C polymorphism). Lastly, the high throughput sequence reads are aligned to final genome type and variants are called to define the intrahost variants of the viral population. For example, the black T would be identified as an intrahost variant.
(1.16 MB TIF)

Figure S5 Scatter plot of ORF depth vs measured nucleotide diversity. For all ORFs, the depth from high throughput sequence data is compared to the calculated nucleotide diversity. The red line represents the linear regression through the data. Equation for linear regression: y = (1.153×10^−5^)x+0.1872. A. The plot is shown for all depth values on a logarithmic scale B. Same plot as in A but only showing depth values between 15–1200, which are the values selected for downstream analysis because the effect of depth on calculated nucleotide diversity is ∼.01%.
(0.45 MB TIF)

Figure S6 HCMV intrahost nucleotide diversity by ORF expression kinetics. Intrahost nucleotide diversity was calculated for each ORF of the HCMV genome. The ORFs were then grouped by expression kinetics and average nucleotide diversity was calculated across all three patients. Error bars represent the 95% confidence interval for the calculated mean. 1-factor ANOVA test for significance: p = 0.0105 (*not significant after Bonferroni correction*).
(2.34 MB TIF)

Figure S7 Unrooted phylogenetic trees of clonal Sanger sequencing of HCMV populations. 3 ORFs per patient sample were selected for clonal Sanger sequencing. The Sanger dataset was then used to generate unrooted phylogenetic trees, using a Jukes-Cantor model of substitution and a neighbor joining method. Scale bars represent substitutions per site. Branch tips are unlabeled except for those representing sequences from the other patient samples, which are highlighted with red text.
(0.35 MB TIF)

Figure S8 Neutral simulations of HCMV populations. 1000 simulations of 3 populations using a standard neutral model were generated via the ms program [24]. Theta and the number of segregating sites in each simulation were matched to the corresponding values from the clinical samples U01, U04 and U33. The simulations were then analyzed using the Sweepfinder program [25] and Composite Likelihood Ratios (CLRs) were generated. The CLRs from the simulations were used to calculate significance thresholds. The 5% significance thresholds for each simulation set are shown as red, dotted lines.
(0.19 MB TIF)

Figure S9 HCMV interhost dN/dS by ORF expression kinetics. Interhost dN/dS values were calculated for each ORF of the HCMV genome. The ORFs were then grouped by expression kinetics and average nucleotide diversity was calculated across all three patients. Error bars represent the 95% confidence interval for the calculated mean. 1-factor ANOVA test for significance: p = 0.1108.
(2.23 MB TIF)

Table S1 Mixed Population Modeling
(0.04 MB DOC)

Table S2 Polymorphism Error Rate for BAC resequencing
(0.04 MB DOC)

Table S3 Whole genome intrahost diversity data from clinical samples
(0.44 MB PDF)

Table S4 Intrahost Nucleotide Diversity Select RNA Viruses
(0.08 MB DOC)

Table S5 ORFs that overlap selective sweeps in the HCMV genome
(0.06 MB PDF)

Table S6 Whole genome interhost polymorphism data from patient samples
(0.27 MB PDF)

Table S7 Genome Wide McDonald-Kreitman Test
(0.19 MB DOC)

Table S8 Primers used in this study to amplify HCMV Genome
(0.15 MB DOC)

Table S9 Assay for Quantitative Capabilities of High Throughput Sequencing Methodology
(0.05 MB DOC)

Text S1 Supporting Figure Legends and References
(0.16 MB DOC)
