# Supplementary material for: Extensive Genome-Wide Variability of Human Cytomegalovirus in Congenitally Infected Infants
Source: PLoS Pathog. 2011 May 19;7(5):e1001344. doi: 10.1371/journal.ppat.1001344 (PMC3098220; doi:10.1371/journal.ppat.1001344)
Supplement: Table S1 — Mixed Population Modeling (0.04 MB DOC) [file ppat.1001344.s010.doc]

**Table S1: Mixed Population Modeling^1^**

| **Depth** | **% Toledo** | **% AD169** | **Variants Detected** | **Detection Rate^2^**  **(%)** | **False Positives^3^** |
| --- | --- | --- | --- | --- | --- |
| 200 | 90 | 10 | 120 | 75.9 | 2 |
| 200 | 99 | 1 | 32 | 20.3 | 2 |
| 200 | 99.5 | 0.5 | 3 | 1.9 | 2 |
| 200 | 99.9 | 0.1 | 3 | 1.9 | 2 |
| 500 | 90 | 10 | 121 | 76.6 | 1 |
| 500 | 99 | 1 | 72 | 45.6 | 1 |
| 500 | 99.5 | 0.5 | 14 | 8.9 | 1 |
| 500 | 99.9 | 0.1 | 3 | 1.9 | 1 |

1. Illumina sequence reads from Toledo and AD169 resequencing experiments were mixed in various ratios, with Toledo being present as the major sequence. These mixes were then aligned to the Toledo genome. Variants were called and thresholds were established that would maximize the detection rate while reducing false positives.

2. Detection Rate is the calculated as the percentage of total known variants (i.e. AD169-derived sequence) that were called by the algorithm.

3. False Positives are the number of called variants that do not represent AD169 sequence.
