# Supplementary material for: Extensive Genome-Wide Variability of Human Cytomegalovirus in Congenitally Infected Infants
Source: PLoS Pathog. 2011 May 19;7(5):e1001344. doi: 10.1371/journal.ppat.1001344 (PMC3098220; doi:10.1371/journal.ppat.1001344)
Supplement: Table S2 — Polymorphism Error Rate for BAC resequencing (0.04 MB DOC) [file ppat.1001344.s011.doc]

**Table S2: Polymorphism Error Rate for BAC resequencing**

| **BAC** | **Polymorphisms** | **Sequenced Region (bp)** | **Error Rate** | **Errors/10 kb** |
| --- | --- | --- | --- | --- |
| **AD169** | 48 | 226372 | 0.021% | 2.1 |
| **Toledo** | 76 | 219628 | 0.035% | 3.5 |
| **Average** | 62 | 223000 | 0.028% | 2.8 |
