# Supplementary material for: Extensive Genome-Wide Variability of Human Cytomegalovirus in Congenitally Infected Infants
Source: PLoS Pathog. 2011 May 19;7(5):e1001344. doi: 10.1371/journal.ppat.1001344 (PMC3098220; doi:10.1371/journal.ppat.1001344)
Supplement: Table S3 — Whole genome intrahost diversity data from clinical samples (0.44 MB PDF) [file ppat.1001344.s012.pdf]

Table S3: Whole genome intrahost diversity data from clinical samples

					U01						U04						U33						Averages					
Feature	Feature Type	Kinetics	Function	Length	Variable Sites	Total Variants	Depth	Nucleotide Diversity	Mean Diversity	Amino Acid Diversity	Variable Sites	Total Variants	Depth	Nucleotide Diversity	Mean Diversity	Amino Acid Diversity	Variable Sites	Total Variants	Depth	Nucleotide Diversity	Mean Diversity	Amino Acid Diversity	Variable Sites	Total Variants	Depth	Nucleotide Diversity	Mean Diversity	Amino Acid Diversity
RL1	gene	unk	Regulatory	933	12	302	151	0.15%	0.18%	0.11%	55	1251	367	0.28%	0.34%	0.18%	26	923	921	0.10%	0.11%	0.07%	31	825	480	0.18%	0.21%	0.12%
RL5A	gene	E	Unknown	288	1	3	69	0.01%	0.01%	0.00%	0	0	79	0.00%	0.00%	0.00%	1	3	69	0.02%	0.02%	0.02%	1	2	72	0.01%	0.01%	0.01%
RL6	gene	L	Unknown	336	0	0	17	0.00%	0.00%	0.00%	0	0	35	0.00%	0.00%	0.00%	0	0	77	0.00%	0.00%	0.00%	0	0	43	0.00%	0.00%	0.00%
RL10	gene	E-L	Glycoprotein	513	6	35	123	0.05%	0.05%	0.04%	5	24	52	0.09%	0.11%	0.06%	13	46	88	0.10%	0.10%	0.08%	8	35	87	0.08%	0.09%	0.06%
RL11	gene	L	Glycoprotein	705	12	3564	891	0.26%	0.57%	0.23%	5	242	830	0.02%	0.02%	0.01%	14	340	393	0.10%	0.10%	0.09%	10	1382	705	0.13%	0.23%	0.11%
RL12	gene	E-L	Unknown	1245	1	3	48	0.01%	0.01%	0.00%	23	214	200	0.09%	0.08%	0.06%	0	0	84	0.00%	0.00%	0.00%	8	72	111	0.03%	0.03%	0.02%
RL13	gene	E-L	Unknown	882	2	9	30	0.03%	0.03%	0.01%	0	0	250	0.00%	0.00%	0.00%	0	0	72	0.00%	0.00%	0.00%	1	3	117	0.01%	0.01%	0.00%
UL1	gene	E-L	Envelope	657	0	0	14	0.00%	0.00%	0.00%	12	96	233	0.06%	0.06%	0.03%	3	349	2379	0.02%	0.02%	0.02%	5	148	875	0.03%	0.03%	0.02%
UL2	gene	L	Unknown	180	8	141	83	0.59%	0.94%	0.47%	1	21	909	0.01%	0.01%	0.00%	2	114	2627	0.02%	0.02%	0.01%	4	92	1206	0.21%	0.33%	0.16%
UL4	gene	E	Glycoprotein	450	15	153	51	0.53%	0.56%	0.39%	0	0	166	0.00%	0.00%	0.00%	5	658	1325	0.12%	0.11%	0.06%	7	270	514	0.22%	0.22%	0.15%
UL5	gene	E	Unknown	501	18	12078	10486	0.21%	0.23%	0.16%	11	498	1508	0.06%	0.07%	0.04%	9	797	2354	0.07%	0.07%	0.03%	13	4458	4783	0.11%	0.12%	0.08%
UL6	gene	unk	Unknown	843	8	347	1594	0.02%	0.03%	0.02%	20	222	351	0.07%	0.07%	0.05%	22	2766	3845	0.09%	0.09%	0.07%	17	1112	1930	0.06%	0.06%	0.05%
UL7	gene	L	Unknown	669	3	46	45	0.10%	0.15%	0.06%	4	27	295	0.01%	0.01%	0.01%	2	252	1828	0.02%	0.02%	0.01%	3	108	723	0.04%	0.06%	0.03%
UL8	gene	unk	Unknown	369	8	3169	8862	0.09%	0.10%	0.05%	13	2229	1371	0.38%	0.44%	0.25%	5	1235	1065	0.11%	0.20%	0.06%	9	2211	3766	0.19%	0.25%	0.12%
UL9	gene	L	Glycoprotein	702	0	0	98	0.00%	0.00%	0.00%	0	0	16	0.00%	0.00%	0.00%	1	3	85	0.01%	0.01%	0.01%	0	1	66	0.00%	0.00%	0.00%
UL10	gene	unk	Unknown	765	0	0	105	0.00%	0.00%	0.00%	8	28	112	0.03%	0.03%	0.01%	0	0	208	0.00%	0.00%	0.00%	3	9	142	0.01%	0.01%	0.00%
UL11	gene	E	Glycoprotein	819	18	517	538	0.10%	0.11%	0.06%	34	409	239	0.20%	0.19%	0.09%	27	1253	814	0.19%	0.19%	0.18%	26	726	530	0.16%	0.16%	0.11%
UL13	gene	E	Unknown	1422	116	64705	9725	0.39%	0.47%	0.26%	167	10319	1171	0.52%	0.62%	0.31%	101	17482	4157	0.31%	0.30%	0.21%	128	30835	5018	0.41%	0.46%	0.26%
UL14	gene	L	Unknown	984	74	2783	1217	0.29%	0.23%	0.25%	108	2166	545	0.51%	0.40%	0.31%	114	7350	2082	0.52%	0.36%	0.42%	99	4100	1281	0.44%	0.33%	0.33%
UL15A	gene	L	Unknown	309	37	3505	2197	0.49%	0.52%	0.36%	44	1669	867	0.64%	0.63%	0.36%	41	3628	2153	0.57%	0.55%	0.43%	41	2934	1739	0.57%	0.56%	0.38%
UL16	gene	E	Glycoprotein	693	28	1247	784	0.21%	0.23%	0.18%	35	1285	622	0.28%	0.30%	0.18%	41	1974	832	0.32%	0.34%	0.28%	35	1502	746	0.27%	0.29%	0.21%
UL17	gene	E	Unknown	315	8	166	704	0.07%	0.08%	0.06%	14	198	495	0.13%	0.13%	0.05%	13	276	723	0.12%	0.12%	0.09%	12	213	641	0.11%	0.11%	0.07%
UL18	gene	L	Glycoprotein	1107	22	1033	463	0.10%	0.17%	0.08%	37	856	385	0.17%	0.19%	0.12%	31	418	279	0.14%	0.12%	0.11%	30	769	376	0.14%	0.16%	0.10%
UL19	gene	unk	Unknown	297	0	0	255	0.00%	0.00%	0.00%	2	72	1538	0.02%	0.02%	0.00%	0	0	255	0.00%	0.00%	0.00%	1	24	683	0.01%	0.01%	0.00%
UL20	gene	unk	Glycoprotein	1017	2	61	774	0.01%	0.01%	0.01%	4	21	164	0.01%	0.01%	0.01%	5	56	220	0.02%	0.01%	0.01%	4	46	386	0.01%	0.01%	0.01%
UL21A	gene	E-L	Glycoprotein	372	14	1181	3272	0.10%	0.10%	0.07%	6	58	290	0.05%	0.05%	0.02%	19	540	537	0.27%	0.27%	0.18%	13	593	1366	0.14%	0.14%	0.09%
UL22A	gene	unk	Glycoprotein	317	5	104	224	0.12%	0.15%	0.08%	24	7113	4962	0.39%	0.42%	0.18%	1	9	149	0.02%	0.02%	0.02%	10	2409	1778	0.17%	0.20%	0.09%
UL23	gene	unk	Matrix/Tegument	855	31	137	109	0.13%	0.13%	0.10%	30	1553	1589	0.11%	0.11%	0.06%	9	30	44	0.08%	0.09%	0.05%	23	573	581	0.11%	0.11%	0.07%
UL24	gene	E-L	Matrix/Tegument	903	38	162	133	0.13%	0.13%	0.12%	29	1760	1704	0.11%	0.11%	0.03%	17	95	52	0.18%	0.19%	0.14%	28	672	630	0.14%	0.15%	0.10%
UL25	gene	L	Matrix/Tegument	1971	24	1286	625	0.09%	0.08%	0.06%	174	71416	5921	0.43%	0.61%	0.17%	78	1599	212	0.27%	0.27%	0.17%	92	24767	2253	0.26%	0.32%	0.14%
UL26	gene	E	Matrix/Tegument	567	17	127	132	0.14%	0.16%	0.11%	104	7066	1906	0.62%	0.65%	0.14%	17	69	43	0.23%	0.23%	0.13%	46	2421	694	0.33%	0.35%	0.13%
UL27	gene	E	Unknown	1827	167	7178	1046	0.35%	0.38%	0.29%	208	19203	1897	0.46%	0.55%	0.22%	174	1181	153	0.41%	0.42%	0.28%	183	9187	1032	0.41%	0.45%	0.26%
UL29	gene	E	Unknown	2102	144	3909	728	0.25%	0.26%	0.19%	142	3457	582	0.28%	0.28%	0.14%	174	1030	136	0.35%	0.35%	0.23%	153	2799	482	0.29%	0.30%	0.19%
UL30	gene	unk	Unknown	366	14	235	650	0.10%	0.10%	0.08%	5	97	564	0.04%	0.05%	0.03%	22	101	133	0.20%	0.20%	0.15%	14	144	449	0.11%	0.12%	0.08%
UL31	gene	L	Unknown	1788	117	23870	4166	0.29%	0.32%	0.24%	133	13060	1908	0.30%	0.38%	0.14%	91	1411	274	0.24%	0.20%	0.17%	114	12780	2116	0.28%	0.30%	0.18%
UL32	gene	L	Matrix/Tegument	3150	205	20715	1388	0.37%	0.47%	0.29%	301	8604	725	0.37%	0.38%	0.19%	134	725	72	0.31%	0.29%	0.22%	213	10015	728	0.35%	0.38%	0.23%
UL33	gene	E	Glycoprotein	1238	29	881	674	0.10%	0.10%	0.08%	38	2796	2067	0.10%	0.11%	0.03%	17	197	226	0.07%	0.06%	0.05%	28	1291	989	0.09%	0.09%	0.05%
UL34	gene	E-L	Regulatory	1224	126	2075	476	0.35%	0.36%	0.27%	112	5160	1314	0.31%	0.32%	0.14%	126	2286	461	0.38%	0.41%	0.28%	121	3174	750	0.35%	0.36%	0.23%
UL35	gene	E	Matrix/Tegument	1926	106	3282	930	0.19%	0.18%	0.15%	119	6941	1708	0.22%	0.21%	0.12%	124	2127	461	0.22%	0.24%	0.18%	116	4117	1033	0.21%	0.21%	0.15%
UL36	gene	IE	Matrix/Tegument	1430	67	878	318	0.18%	0.19%	0.14%	41	1542	958	0.10%	0.11%	0.06%	37	1254	873	0.09%	0.10%	0.07%	48	1225	717	0.13%	0.13%	0.09%
UL37	gene	IE	Regulatory	1465	46	274	220	0.21%	0.19%	0.10%	79	762	219	0.34%	0.34%	0.09%	10	36	67	0.06%	0.06%	0.05%	45	357	169	0.20%	0.19%	0.08%
UL38	gene	IE	Unknown	996	37	530	127	0.12%	0.12%	0.16%	58	2171	428	0.17%	0.34%	0.16%	17	166	72	0.10%	0.11%	0.04%	37	956	209	0.13%	0.19%	0.12%
UL40	gene	E-L	Regulatory	666	55	819	330	0.36%	0.37%	0.24%	57	857	320	0.38%	0.40%	0.16%	42	11072	6186	0.26%	0.27%	0.17%	51	4249	2279	0.33%	0.35%	0.19%
UL41A	gene	L	Unknown	237	14	106	217	0.21%	0.21%	0.17%	11	113	207	0.24%	0.23%	0.16%	7	842	3278	0.13%	0.11%	0.09%	11	354	1234	0.19%	0.18%	0.14%
UL42	gene	unk	Unknown	378	25	325	340	0.24%	0.25%	0.18%	27	313	309	0.25%	0.27%	0.11%	19	3657	4653	0.20%	0.21%	0.17%	24	1432	1767	0.23%	0.24%	0.15%
UL43	gene	L	Matrix/Tegument	1272	178	9054	1187	0.57%	0.60%	0.43%	194	10982	1203	0.63%	0.63%	0.29%	122	8963	1525	0.42%	0.43%	0.30%	165	9666	1305	0.54%	0.55%	0.34%
UL44	gene	E-L	DNA Replication	1302	76	558	167	0.25%	0.26%	0.12%	86	2927	707	0.29%	0.32%	0.10%	22	101	54	0.17%	0.18%	0.05%	61	1195	310	0.23%	0.25%	0.09%
UL45	gene	L	Matrix/Tegument	2721	122	2140	534	0.16%	0.15%	0.13%	185	12793	1964	0.24%	0.24%	0.12%	226	2215	257	0.32%	0.31%	0.22%	178	5716	918	0.24%	0.23%	0.16%
UL46	gene	E-L	capsid	2952	146	24194	859	0.18%	0.16%	0.14%	190	3950	407	0.23%	0.32%	0.10%	183	6675	1078	0.21%	0.20%	0.20%	173	11606	782	0.21%	0.23%	0.15%
UL47	gene	E-L	Matrix/Tegument	873	43	1371	5050	0.17%	0.18%	0.14%	42	3078	2396	0.14%	0.15%	0.12%	69	467	191	0.28%	0.28%	0.15%	51	1639	2546	0.20%	0.20%	0.14%
UL48	gene	L	capsid	6726	385	35080	1659	0.21%	0.31%	0.16%	422	2573	130	0.26%	0.26%	0.13%	483	15812	748	0.30%	0.31%	0.23%	430	17822	846	0.26%	0.29%	0.17%
UL48A	gene	unk	capsid	228	22	354	497	0.30%	0.31%	0.28%	4	14	49	0.12%	0.12%	0.06%	25	499	293	0.62%	0.62%	0.54%	17	289	280	0.35%	0.35%	0.29%
UL49	gene	E-L	capsid	1713	193	6002	797	0.43%	0.44%	0.35%	210	2516	209	0.54%	0.66%	0.25%	186	5873	744	0.44%	0.46%	0.34%	196	4797	583	0.47%	0.52%	0.31%
UL50	gene	unk	Nuclear Egress	1197	68	4045	927	0.25%	0.35%	0.20%	89	1013	321	0.28%	0.25%	0.11%	95	6038	1741	0.36%	0.29%	0.25%	84	3699	996	0.30%	0.29%	0.18%
UL51	gene	unk	DNA Packaging	474	15	87	63	0.40%	0.43%	0.19%	31	2413	1437	0.34%	0.36%	0.19%	36	5807	3339	0.34%	0.37%	0.24%	27	2769	1613	0.36%	0.38%	0.21%
UL52	gene	L	DNA Packaging	2007	50	224	49	0.21%	0.23%	0.12%	126	5828	1040	0.26%	0.28%	0.14%	108	10704	2491	0.21%	0.21%	0.16%	95	5585	1194	0.23%	0.24%	0.14%
UL53	gene	E	Nuclear Egress	1131	73	1899	494	0.32%	0.30%	0.24%	103	13676	3876	0.34%	0.31%	0.17%	85	11257	3146	0.28%	0.32%	0.21%	87	8944	2505	0.31%	0.31%	0.21%
UL54	gene	E	DNA Replication	3729	141	9879	1255	0.17%	0.21%	0.13%	229	22801	1375	0.27%	0.44%	0.13%	73	4196	1486	0.11%	0.07%	0.07%	148	12292	1372	0.18%	0.24%	0.11%
UL55	gene	E	Glycoprotein	2724	71	5001	1785	0.10%	0.10%	0.07%	105	2484	548	0.15%	0.16%	0.08%	102	5603	1376	0.15%	0.15%	0.10%	93	4363	1236	0.13%	0.14%	0.08%
UL56	gene	E	DNA Packaging	2553	102	13290	3353	0.15%	0.16%	0.10%	124	834	141	0.20%	0.22%	0.11%	108	2290	467	0.17%	0.19%	0.10%	111	5471	1320	0.17%	0.19%	0.10%
UL57	gene	E	DNA Replication	3708	116	1876	163	0.20%	0.20%	0.16%	280	3174	218	0.32%	0.36%	0.14%	209	4739	608	0.21%	0.21%	0.16%	202	3263	330	0.24%	0.26%	0.15%
UL69	gene	E-L	Regulatory	2229	82	9701	3054	0.16%	0.14%	0.13%	74	14703	5958	0.11%	0.11%	0.06%	85	2570	657	0.16%	0.17%	0.12%	80	8991	3223	0.15%	0.14%	0.11%
UL70	gene	E-L	DNA Replication	2841	147	8477	1251	0.23%	0.24%	0.19%	247	22753	2225	0.35%	0.36%	0.19%	214	5685	554	0.34%	0.36%	0.26%	203	12305	1343	0.31%	0.32%	0.21%
UL71	gene	unk	Unknown	1086	87	4144	1184	0.31%	0.32%	0.26%	118	8739	1949	0.40%	0.41%	0.20%	108	2354	533	0.38%	0.41%	0.30%	104	5079	1222	0.36%	0.38%	0.25%
UL72	gene	L	Unknown	1167	42	7264	3215	0.17%	0.19%	0.13%	54	4118	2172	0.17%	0.16%	0.08%	56	1903	1144	0.17%	0.14%	0.10%	51	4428	2177	0.17%	0.17%	0.10%
UL73	gene	E-L	Glycoprotein	408	8	2556	1375	0.19%	0.37%	0.16%	0	0	183	0.00%	0.00%	0.00%	7	316	463	0.05%	0.15%	0.02%	5	957	674	0.08%	0.17%	0.06%
UL74	gene	unk	Glycoprotein	1419	6	62	172	0.02%	0.03%	0.02%	21	469	273	0.11%	0.11%	0.05%	4	17	52	0.02%	0.02%	0.02%	10	183	166	0.05%	0.05%	0.03%
UL75	gene	E-L	Glycoprotein	2229	33	1554	957	0.08%	0.05%	0.07%	64	6945	2947	0.13%	0.11%	0.07%	57	338	91	0.15%	0.15%	0.09%	51	2946	1331	0.12%	0.10%	0.08%
UL76	gene	unk	Regulatory	978	45	212	95	0.21%	0.22%	0.14%	73	4311	1350	0.31%	0.33%	0.15%	36	198	60	0.25%	0.34%	0.16%	51	1574	501	0.26%	0.29%	0.15%
UL77	gene	unk	DNA Packaging	1911	114	4073	423	0.31%	0.41%	0.23%	174	41395	4188	0.40%	0.52%	0.18%	110	7556	761	0.32%	0.36%	0.22%	133	17675	1790	0.34%	0.43%	0.21%
UL78	gene	E	Regulatory	1296	44	403	224	0.13%	0.14%	0.11%	70	4312	1742	0.18%	0.19%	0.10%	64	1939	743	0.19%	0.20%	0.16%	59	2218	903	0.17%	0.18%	0.12%
UL79	gene	E-L	Unknown	888	45	315	172	0.19%	0.19%	0.17%	55	2205	1312	0.18%	0.19%	0.11%	59	1121	509	0.24%	0.25%	0.21%	53	1214	665	0.20%	0.21%	0.16%
UL80	gene	L	capsid	2127	169	6166	498	0.38%	0.48%	0.27%	265	21090	1948	0.49%	0.50%	0.19%	241	9193	953	0.48%	0.45%	0.35%	225	12150	1133	0.45%	0.48%	0.27%
UL80.5	gene	unk	capsid	1122	72	3572	642	0.34%	0.33%	0.19%	152	12877	1967	0.54%	0.57%	0.20%	144	5546	925	0.58%	0.53%	0.38%	123	7332	1178	0.48%	0.48%	0.26%
UL82	gene	L	Matrix/Tegument	1680	125	1840	343	0.31%	0.30%	0.18%	120	2028	502	0.24%	0.24%	0.13%	93	5779	1757	0.20%	0.20%	0.14%	113	3216	867	0.25%	0.25%	0.15%
UL83	gene	L	Matrix/Tegument	1686	89	3021	850	0.20%	0.21%	0.17%	146	873	135	0.37%	0.37%	0.18%	52	8883	3459	0.14%	0.15%	0.11%	96	4259	1481	0.24%	0.24%	0.15%

UL84	gene	E-L	DNA Replication	1764	171	5809	806	0.40%	0.41%	0.29%	155	1176	154	0.39%	0.41%	0.15%	143	40175	4413	0.40%	0.52%	0.23%	156	15720	1791	0.40%	0.45%	0.22%
UL85	gene	E-L	capsid	921	61	2898	1180	0.26%	0.27%	0.21%	72	1066	303	0.37%	0.38%	0.19%	67	13896	4218	0.32%	0.36%	0.24%	67	5953	1900	0.32%	0.34%	0.21%
UL86	gene	E-L	capsid	4113	219	6781	717	0.22%	0.23%	0.15%	180	3954	624	0.15%	0.15%	0.07%	173	19960	3466	0.14%	0.14%	0.09%	191	10232	1602	0.17%	0.17%	0.11%
UL87	gene	E-L	Matrix?	2826	163	2065	204	0.29%	0.30%	0.21%	196	7063	882	0.28%	0.28%	0.13%	227	65160	5463	0.33%	0.42%	0.25%	195	24763	2183	0.30%	0.34%	0.20%
UL88	gene	unk	Matrix/Tegument	1290	95	2109	279	0.40%	0.52%	0.26%	131	4059	570	0.45%	0.55%	0.22%	101	9266	2465	0.27%	0.29%	0.17%	109	5145	1104	0.37%	0.45%	0.22%
UL89	gene	E-L	DNA Packaging	2024	38	351	241	0.22%	0.23%	0.18%	47	544	224	0.25%	0.26%	0.18%	36	5551	254	0.18%	0.18%	0.21%	40	2149	239	0.22%	0.22%	0.19%
UL91	gene	L	Unknown	606	107	7341	204	0.28%	0.30%	0.09%	158	9269	273	0.39%	0.49%	0.13%	113	29050	6886	0.26%	0.24%	0.12%	126	15220	2454	0.31%	0.34%	0.12%
UL92	gene	L	Unknown	1785	38	638	254	0.16%	0.17%	0.18%	39	672	352	0.16%	0.17%	0.13%	48	7198	5193	0.20%	0.21%	0.15%	42	2836	1933	0.17%	0.18%	0.15%
UL93	gene	L	DNA Packaging	1038	125	2271	1350	0.34%	0.29%	0.19%	126	1813	1047	0.35%	0.28%	0.20%	51	26175	6760	0.21%	0.23%	0.17%	101	10086	3052	0.30%	0.26%	0.19%
UL94	gene	L	Matrix/Tegument	1602	99	2467	358	0.23%	0.24%	0.14%	428	13225	385	0.30%	0.36%	0.07%	342	75348	3282	0.23%	0.22%	0.15%	290	30347	1342	0.25%	0.27%	0.12%
UL95	gene	E-L	Matrix?	5928	325	11107	462	0.25%	0.27%	0.23%	148	2467	393	0.32%	0.30%	0.16%	132	31463	5438	0.27%	0.27%	0.17%	202	15012	2097	0.28%	0.28%	0.18%
UL96	gene	E-L	Matrix/Tegument	384	19	115	513	0.16%	0.16%	0.15%	16	95	396	0.11%	0.11%	0.06%	18	90	5806	0.13%	0.13%	0.09%	18	100	2238	0.13%	0.13%	0.10%
UL97	gene	E-L	Matrix/Tegument	2124	140	1498	187	0.29%	0.26%	0.21%	166	1986	222	0.34%	0.27%	0.14%	182	7330	183	0.36%	0.36%	0.27%	163	3605	197	0.33%	0.30%	0.21%
UL98	gene	E-L	DNA Replication	1755	155	1199	247	0.36%	0.35%	0.25%	193	1983	332	0.44%	0.47%	0.22%	200	16663	939	0.51%	0.47%	0.36%	183	6615	506	0.44%	0.43%	0.28%
UL99	gene	L	Envelope	573	33	597	189	0.20%	0.24%	0.16%	50	1578	238	0.38%	0.42%	0.21%	41	7496	2025	0.34%	0.53%	0.20%	41	3224	817	0.31%	0.40%	0.19%
UL100	gene	E-L	Glycoprotein	1116	0	0	405	0.00%	0.00%	0.00%	51	2808	657	0.17%	0.17%	0.08%	25	129	2469	0.15%	0.14%	0.08%	25	979	1177	0.11%	0.10%	0.05%
UL102	gene	L	DNA Replication	2622	0	0	34	0.00%	0.00%	0.00%	334	3376	1486	0.59%	0.62%	0.23%	112	558	77	0.40%	0.39%	0.26%	149	1311	533	0.33%	0.34%	0.16%
UL103	gene	L	Matrix/Tegument	750	0	0	7	0.00%	0.00%	0.00%	33	276	207	0.13%	0.14%	0.09%	14	53	54	0.09%	0.09%	0.07%	16	110	89	0.07%	0.08%	0.05%
UL104	gene	E	capsid	2871	97	814	7	0.18%	0.14%	0.07%	207	61019	264	0.28%	0.22%	0.16%	253	21232	77	0.34%	0.34%	0.08%	186	27688	116	0.27%	0.23%	0.10%
UL105	gene	E	DNA Replication	2094	26	190	117	0.08%	0.07%	0.13%	169	11204	255	0.31%	0.39%	0.15%	43	1843	255	0.14%	0.07%	0.25%	79	4412	209	0.17%	0.18%	0.18%
UL111A	gene	E-L	Immune Evasion	183	0	0	147	0.00%	0.00%	0.00%	36	635	9619	0.38%	0.35%	0.03%	0	0	2179	0.00%	0.00%	0.00%	12	212	3982	0.13%	0.12%	0.01%
UL112	gene	E	DNA Replication	2054	65	696	96	0.15%	0.17%	0.10%	320	9543	1380	0.64%	0.64%	0.35%	19	135	649	0.13%	0.15%	0.05%	135	3458	709	0.31%	0.32%	0.16%
UL114	gene	E	DNA Replication	753	10	37	31	0.07%	0.08%	0.06%	70	2200	348	0.34%	0.33%	0.17%	0	0	3	0.00%	0.00%	0.00%	27	746	127	0.14%	0.14%	0.08%
UL115	gene	L	Glycoprotein	837	26	210	175	0.18%	0.16%	0.16%	66	2671	624	0.29%	0.30%	0.15%	35	261	54	0.22%	0.21%	0.19%	42	1047	284	0.23%	0.22%	0.17%
UL116	gene	E-L	Unknown	942	6	57	62	0.02%	0.02%	0.02%	57	3052	884	0.34%	0.32%	0.16%	34	190	17	0.22%	0.17%	0.17%	32	1100	321	0.19%	0.17%	0.11%
UL117	gene	L	Unknown	1278	1	3	116	0.01%	0.01%	0.00%	149	5396	1082	0.64%	0.61%	0.29%	50	198	139	0.29%	0.22%	0.17%	67	1866	446	0.31%	0.28%	0.15%
UL119	gene	E	Glycoprotein	1037	21	133	203	0.12%	0.09%	0.11%	11	890	1001	0.04%	0.02%	0.01%	14	84	100	0.14%	0.12%	0.09%	15	369	435	0.10%	0.08%	0.07%
UL120	gene	L	Unknown	606	2	9	22	0.03%	0.03%	0.03%	10	359	691	0.08%	0.10%	0.02%	3	10	67	0.02%	0.02%	0.02%	5	126	260	0.04%	0.05%	0.02%
UL121	gene	L	Unknown	543	21	109	117	0.19%	0.20%	0.15%	22	927	3497	0.16%	0.17%	0.07%	22	145	73	0.18%	0.20%	0.14%	22	394	1229	0.18%	0.19%	0.12%
UL122	gene	IE,L	Regulatory	1741	8	91	67	0.02%	0.02%	0.08%	18	586	605	0.03%	0.04%	0.12%	12	229	86	0.03%	0.04%	0.11%	13	302	252	0.03%	0.03%	0.10%
UL123	gene	IE	Regulatory	1474	32	153	92	0.11%	0.11%	0.01%	102	3671	985	0.23%	0.24%	0.02%	54	311	126	0.14%	0.15%	0.02%	63	1378	401	0.16%	0.17%	0.02%
UL124	gene	E	Unknown	453	53	1102	75	0.53%	0.48%	0.46%	48	847	885	0.37%	0.39%	0.21%	57	1059	106	0.55%	0.57%	0.46%	53	1003	355	0.48%	0.48%	0.38%
UL128	gene	E	Glycoprotein	391	22	795	213	0.23%	0.25%	0.20%	18	227	1105	0.22%	0.22%	0.09%	21	1930	336	0.21%	0.23%	0.18%	20	984	551	0.22%	0.23%	0.16%
UL130	gene	E-L	Glycoprotein	645	10	462	507	0.04%	0.05%	0.03%	18	241	481	0.08%	0.09%	0.03%	7	863	411	0.04%	0.04%	0.03%	12	522	466	0.06%	0.06%	0.03%
UL131A	gene	L	Glycoprotein	389	34	7296	800	0.35%	0.27%	0.31%	28	420	270	0.41%	0.44%	0.29%	24	2841	2147	0.30%	0.28%	0.24%	29	3519	1072	0.35%	0.33%	0.28%
UL132	gene	E-L	Glycoprotein	813	15	1283	1588	0.07%	0.08%	0.05%	18	243	433	0.10%	0.11%	0.03%	19	1889	3012	0.09%	0.10%	0.06%	17	1138	1677	0.09%	0.09%	0.05%
UL148	gene	unk	Unknown	951	33	4977	6865	0.11%	0.12%	0.09%	38	1320	244	0.14%	0.12%	0.09%	32	8989	2571	0.14%	0.13%	0.10%	34	5095	3227	0.13%	0.12%	0.09%
UL147A	gene	unk	Unknown	228	0	0	2018	0.00%	0.00%	0.00%	0	0	278	0.00%	0.00%	0.00%	0	0	2397	0.00%	0.00%	0.00%	0	0	1564	0.00%	0.00%	0.00%
UL147	gene	E-L	Regulatory	480	0	0	4449	0.00%	0.00%	0.00%	1	144	1183	0.01%	0.01%	0.01%	2	25	7394	0.02%	0.02%	0.01%	1	56	4342	0.01%	0.01%	0.01%
UL146	gene	unk	Regulatory	363	0	0	255	0.00%	0.00%	0.00%	0	0	2105	0.00%	0.00%	0.00%	0	0	227	0.00%	0.00%	0.00%	0	0	862	0.00%	0.00%	0.00%
UL145	gene	unk	Unknown	393	1	28	1162	0.01%	0.01%	0.00%	1	18	672	0.01%	0.01%	0.01%	1	61	1975	0.01%	0.01%	0.00%	1	36	1270	0.01%	0.01%	0.00%
UL144	gene	unk	Glycoprotein	531	0	0	252	0.00%	0.00%	0.00%	1	5	103	0.01%	0.01%	0.01%	4	1914	922	0.16%	0.27%	0.07%	2	640	426	0.06%	0.09%	0.03%
UL142	gene	unk	Glycoprotein	918	4	50	357	0.02%	0.02%	0.01%	10	94	211	0.05%	0.05%	0.03%	18	248	288	0.09%	0.09%	0.07%	11	131	285	0.05%	0.05%	0.04%
UL141	gene	unk	Glycoprotein	1017	47	7599	3205	0.23%	0.22%	0.14%	72	7571	2847	0.38%	0.26%	0.21%	81	14368	3483	0.44%	0.41%	0.31%	67	9846	3178	0.35%	0.30%	0.22%
UL140	gene	unk	Unknown	576	3	16	46	0.06%	0.06%	0.05%	40	4782	3009	0.26%	0.28%	0.14%	83	2567	759	0.53%	0.59%	0.46%	42	2455	1271	0.29%	0.31%	0.22%
UL139	gene	unk	Glycoprotein	417	0	0	21	0.00%	0.00%	0.00%	0	0	190	0.00%	0.00%	0.00%	20	435	339	0.26%	0.30%	0.20%	7	145	183	0.09%	0.10%	0.07%
UL138	gene	unk	Regulatory	510	1	4	39	0.01%	0.01%	0.00%	26	3347	3576	0.17%	0.18%	0.09%	17	628	886	0.13%	0.14%	0.12%	15	1326	1500	0.10%	0.11%	0.07%
UL136	gene	unk	Unknown	723	0	0	17	0.00%	0.00%	0.00%	106	10204	2746	0.50%	0.51%	0.25%	123	2970	641	0.61%	0.64%	0.49%	76	4391	1134	0.37%	0.39%	0.25%
UL135	gene	unk	Unknown	927	5	18	26	0.07%	0.07%	0.06%	133	10466	2044	0.53%	0.55%	0.24%	132	6095	1146	0.59%	0.57%	0.41%	90	5526	1072	0.40%	0.40%	0.24%
UL133	gene	unk	Unknown	774	0	0	5	0.00%	0.00%	0.00%	76	6471	2021	0.39%	0.41%	0.18%	79	3101	833	0.43%	0.48%	0.27%	52	3191	953	0.27%	0.30%	0.15%
UL148A	gene	unk	Unknown	243	8	191	874	0.08%	0.09%	0.08%	13	778	1060	0.30%	0.30%	0.08%	6	136	135	0.22%	0.34%	0.11%	9	368	690	0.20%	0.24%	0.09%
UL148B	gene	unk	Unknown	243	7	162	657	0.10%	0.10%	0.05%	8	425	1326	0.13%	0.13%	0.09%	0	0	19	0.00%	0.00%	0.00%	5	196	667	0.08%	0.08%	0.05%
UL148C	gene	unk	Unknown	234	22	332	484	0.29%	0.29%	0.25%	26	1560	1119	0.54%	0.60%	0.29%	1	3	15	0.07%	0.09%	0.01%	16	632	539	0.30%	0.32%	0.18%
UL148D	gene	unk	Unknown	189	1	24	646	0.02%	0.02%	0.02%	2	55	1357	0.02%	0.02%	0.02%	1	3	28	0.05%	0.06%	0.05%	1	27	677	0.03%	0.03%	0.03%
UL150	gene	unk	Unknown	1920	85	34608	11013	0.17%	0.16%	0.13%	103	50396	16047	0.17%	0.16%	0.08%	127	246289	15033	0.29%	0.85%	0.20%	105	110431	14031	0.21%	0.39%	0.14%
IRS1	gene	IE	Regulatory	2544	58	9132	4000	0.10%	0.09%	0.06%	41	23230	7661	0.08%	0.12%	0.04%	30	564	164	0.09%	0.12%	0.05%	43	10975	3942	0.09%	0.11%	0.05%
US1	gene	unk	Unknown	471	54	7157	3499	0.41%	0.44%	0.34%	63	15455	6074	0.51%	0.54%	0.28%	5	19	48	0.07%	0.07%	0.05%	41	7544	3207	0.33%	0.35%	0.23%
US2	gene	E	Immune Evasion	600	13	1051	1035	0.15%	0.17%	0.11%	10	2079	1806	0.16%	0.19%	0.10%	11	199	85	0.28%	0.32%	0.14%	11	1110	975	0.19%	0.23%	0.12%
US3	gene	IE	Immune Evasion	561	10	223	474	0.08%	0.08%	0.06%	13	862	1173	0.13%	0.13%	0.07%	1	3	18	0.03%	0.04%	0.00%	8	363	555	0.08%	0.08%	0.04%
US6	gene	E-L	Immune Evasion	549	13	320	417	0.13%	0.14%	0.12%	3	96	1160	0.01%	0.02%	0.00%	1	15	148	0.02%	0.02%	0.02%	6	144	575	0.05%	0.06%	0.05%
US7	gene	E-L	Immune Evasion	678	28	2142	1626	0.18%	0.19%	0.16%	36	1126	664	0.24%	0.25%	0.12%	28	791	578	0.19%	0.20%	0.13%	31	1353	956	0.20%	0.21%	0.14%
US8	gene	E	Immune Evasion	684	41	7565	3991	0.36%	0.25%	0.29%	35	5679	2503	0.35%	0.33%	0.15%	41	3224	1305	0.43%	0.35%	0.38%	39	5489	2600	0.38%	0.31%	0.28%
US9	gene	E	Immune Evasion	744	34	143	118	0.16%	0.15%	0.13%	36	2792	2109	0.17%	0.18%	0.11%	37	482	179	0.28%	0.25%	0.20%	36	1139	802	0.20%	0.19%	0.14%
US10	gene	E	Immune Evasion	558	14	56	65	0.18%	0.17%	0.10%	43	2713	1406	0.32%	0.35%	0.16%	11	45	44	0.16%	0.18%	0.14%	23	938	505	0.22%	0.23%	0.14%
US11	gene	E	Immune Evasion	648	6	24	77	0.05%	0.05%	0.04%	12	436	1218	0.05%	0.06%	0.02%	6	33	89	0.04%	0.04%	0.03%	8	164	461	0.05%	0.05%	0.03%
US12	gene	E	Unknown	846	35	892	338	0.25%	0.28%	0.14%	31	5519	4317	0.19%	0.15%	0.09%	29	283	113	0.24%	0.25%	0.11%	32	2231	1589	0.23%	0.23%	0.12%
US13	gene	E	Unknown	786	21	804	871	0.11%	0.12%	0.08%	29	2983	2521	0.15%	0.15%	0.07%	14	69	73	0.12%	0.11%	0.04%	21	1285	1155	0.12%	0.13%	0.06%
US14	gene	E	Unknown	933	33	1258	1040	0.12%	0.13%	0.11%	41	4249	2780	0.15%	0.16%	0.11%	50	529	231	0.25%	0.22%	0.14%	41	2012	1350	0.17%	0.17%	0.12%
US15	gene	E-L	Unknown	789	21	545	957	0.07%	0.07%	0.06%	33	2756	2296	0.15%	0.15%	0.08%	17	115	93	0.13%	0.13%	0.08%	24	1139	1115	0.11%	0.12%	0.07%
US16	gene	E	Unknown	930	45	8016	2795	0.25%	0.31%	0.20%	70	11790	2725	0.40%	0.47%	0.18%	67	8438	1590	0.47%	0.51%	0.36%	61	9415	2370	0.37%	0.43%	0.25%
US17	gene	E	Unknown	882	25	667	564	0.13%	0.13%	0.11%	39	1807	1044	0.19%	0.20%	0.11%	33	624	579	0.12%	0.12%	0.10%	32	1033	729	0.14%	0.15%	0.11%
US18	gene	E	Unknown	825	26	1856	1207	0.15%	0.19%	0.14%	45	2947	1554	0.22%	0.23%	0.16%	40	2088	1083	0.21%	0.23%	0.19%	37	2297	1281	0.20%	0.22%	0.16%
US19	gene	E	Unknown	723	60	3711	1216	0.36%	0.42%	0.28%	86	4373	1120	0.51%	0.54%	0.26%	73	2277	732	0.40%	0.43%	0.32%	73	3454	1023	0.43%	0.46%	0.29%
US20	gene	E	Unknown	765	25	889	895	0.12%	0.13%	0.09%	29	1433	1174	0.15%	0.16%	0.09%	25	712	715	0.12%	0.13%	0.10%	26	1011	928	0.13%	0.14%	0.09%
US21	gene	unk	Unknown	732	23	627	458	0.21%	0.19%	0.16%	35	1096	551	0.27%	0.27%	0.14%	36	886	486	0.25%	0.25%	0.24%	31	870	498	0.24%	0.24%	0.18%
US22	gene	E	Matrix/Tegument	1731	55	3275	1588	0.12%	0.11%	0.08%	53	2742	1483	0.11%	0.11%	0.06%	54	1542	522	0.13%	0.14%	0.08%	54	2520	1198	0.12%	0.12%	0.07%
US23	gene	E	Matrix/Tegument	1779	38	141	71	0.11%	0.11%	0.08%	92	4040	1466	0.15%	0.16%	0.07%	84	530	149	0.17%	0.19%	0.12%	71	1570	562	0.14%	0.15%	0.09%
US24	gene	E	Matrix/Tegument	1506	26	97	74	0.09%	0.09%	0.06%	74	3257	1239	0.17%	0.17%	0.09%	49	302	127	0.14%	0.15%	0.08%	50	1219	480	0.13%	0.14%	0.08%
US26	gene	E	Unknown	1812	10	39	44	0.06%	0.06%	0.03%	64	2290	862	0.15%	0.15%	0.10%	37	200	86	0.13%	0.12%	0.09%	37	843	331	0.11%	0.11%	0.08%
US27	gene	E	Glycoprotein	1095	10	63	78	0.06%	0.07%	0.05%	10	733	1502	0.04%	0.04%	0.02%	22	683	734	0.10%	0.09%	0.05%	14	493	771	0.07%	0.07%	0.04%
US28	gene	E	Envelope	1065	28	113	108	0.09%	0.09%	0.07%	17	826	1512	0.05%	0.05%	0.02%	32	1184	773	0.13%	0.14%	0.11%	26	708	798	0.09%	0.10%	0.07%
US29	gene	E-L	Unknown	1389	127	22207	3014	0.44%	0.49%	0.32%	123	41475	4484	0.45%	0.65%	0.19%	134	50792	7841	0.44%	0.47%	0.31%	128	38158	5113	0.44%	0.53%	0.27%

US30	gene	E	Unknown	1050	31	661	554	0.11%	0.11%	0.09%	0	0	20	0.00%	0.00%	0.00%	21	784	1175	0.06%	0.06%	0.05%	17	482	583	0.06%	0.06%	0.05%
US31	gene	unk	Unknown	486	28	812	561	0.29%	0.30%	0.25%	0	0	5	0.00%	0.00%	0.00%	41	1842	1026	0.35%	0.37%	0.29%	23	885	531	0.21%	0.22%	0.18%
US32	gene	L	Unknown	552	42	858	577	0.26%	0.27%	0.23%	0	0	5	0.00%	0.00%	0.00%	42	1893	982	0.33%	0.35%	0.27%	28	917	521	0.20%	0.21%	0.16%
US34	gene	E	Unknown	492	27	650	581	0.24%	0.23%	0.22%	44	1363	730	0.36%	0.38%	0.21%	23	1298	1461	0.17%	0.18%	0.16%	31	1104	924	0.26%	0.26%	0.20%
US34A	gene	unk	Unknown	195	1	9	370	0.01%	0.01%	0.01%	1	10	523	0.01%	0.01%	0.01%	0	0	255	0.00%	0.00%	0.00%	1	6	383	0.01%	0.01%	0.01%
TRS1	gene	E	Regulatory	2367	54	4688	1108	0.14%	0.17%	0.09%	58	8772	3297	0.11%	0.11%	0.05%	53	15080	5980	0.11%	0.11%	0.08%	55	9513	3462	0.12%	0.13%	0.08%
UL112	miRNA			67	0	0	72	0.00%	0.00%	NA	0	0	1124	0.00%	0.00%	NA	0	0	53	0.00%	0.00%	NA	0	0	416	0.00%	0.00%	NA
UL148D	miRNA			72	1	4	3960	0.03%	0.03%	NA	0	0	10077	0.00%	0.00%	NA	0	0	2656	0.00%	0.00%	NA	0	1	5564	0.01%	0.01%	NA
UL22A	miRNA			68	5	184	221	0.22%	0.22%	NA	12	1305	4483	0.91%	0.99%	NA	0	0	147	0.00%	0.00%	NA	6	496	1617	0.38%	0.40%	NA
UL36	miRNA			75	1	4	187	0.07%	0.07%	NA	0	0	944	0.00%	0.00%	NA	0	0	875	0.00%	0.00%	NA	0	1	669	0.02%	0.02%	NA
UL70	miRNA			62	5	550	1349	0.19%	0.20%	NA	5	1798	2167	0.24%	0.25%	NA	8	757	544	0.39%	0.40%	NA	6	1035	1353	0.27%	0.28%	NA
US25-1	miRNA			70	3	39	33	0.10%	0.10%	NA	1	30	612	0.03%	0.03%	NA	0	0	27	0.00%	0.00%	NA	1	23	224	0.04%	0.04%	NA
US25-2	miRNA			90	0	0	61	0.00%	0.00%	NA	1	137	642	0.04%	0.04%	NA	1	25	91	0.04%	0.04%	NA	1	54	265	0.03%	0.03%	NA
US33	miRNA			70	0	0	100	0.00%	0.00%	NA	0	0	1459	0.00%	0.00%	NA	0	0	1321	0.00%	0.00%	NA	0	0	960	0.00%	0.00%	NA
US4	miRNA			84	3	11	476	0.45%	0.50%	NA	16	476	1369	1.06%	1.13%	NA	0	0	22	0.00%	0.00%	NA	6	162	622	0.50%	0.54%	NA
US5-1	miRNA			66	0	0	431	0.00%	0.00%	NA	3	46	5060	0.08%	0.08%	NA	0	0	928	0.00%	0.00%	NA	1	15	2140	0.03%	0.03%	NA
US5-2	miRNA			65	15	94	433	1.19%	1.34%	NA	19	1249	5067	1.17%	1.24%	NA	25	2386	908	2.17%	2.62%	NA	20	1243	2136	1.51%	1.73%	NA
UL	Misc	NA	NA	193019	7268	463036	1201	0.17%	0.18%	NA	11679	704159	1347	0.26%	0.26%	NA	8964	843029	1528	0.21%	0.25%	NA	9304	670075	1358	0.21%	0.23%	NA
US	Misc	NA	NA	35482	1248	98620	1409	0.16%	0.17%	NA	1615	170137	2178	0.20%	0.22%	NA	1329	100502	1312	0.19%	0.18%	NA	1397	123086	1633	0.18%	0.19%	NA
TRL	Misc	NA	NA	1324	8	256	882	0.02%	0.02%	NA	1	198	306	0.02%	0.03%	NA	2	47	26	0.01%	0.14%	NA	4	167	404	0.02%	0.06%	NA
IRL	Misc	NA	NA	1324	8	1142	1870	0.04%	0.04%	NA	23	5533	6815	0.07%	0.06%	NA	8	420	143	0.08%	0.22%	NA	13	2365	2943	0.07%	0.11%	NA
IRS	Misc	NA	NA	2537	13	855	420	0.03%	0.07%	NA	22	18941	8263	0.06%	0.09%	NA	7	416	155	0.04%	0.11%	NA	14	6737	2946	0.04%	0.09%	NA
TRS	Misc	NA	NA	2538	20	691	494	0.08%	0.05%	NA	10	2361	1465	0.04%	0.06%	NA	15	1378	1142	0.06%	0.05%	NA	15	1477	1034	0.06%	0.05%	NA
