# Supplementary material for: Extensive Genome-Wide Variability of Human Cytomegalovirus in Congenitally Infected Infants
Source: PLoS Pathog. 2011 May 19;7(5):e1001344. doi: 10.1371/journal.ppat.1001344 (PMC3098220; doi:10.1371/journal.ppat.1001344)
Supplement: Table S4 — Intrahost Nucleotide Diversity Select RNA Viruses (0.08 MB DOC) [file ppat.1001344.s013.doc]

**Table S4: Intrahost Nucleotide Diversity Select RNA Viruses**

| **Virus** | **Intrahost**  **Nucleotide Diversity (%)** | **Reference** |
| --- | --- | --- |
| infectious hematopoetic necrosis virus | 0.001 |  |
| West Nile virus | 0.03 |  |
| human immunodeficiency virus-1 (min)^1^ | 0.04 |  |
| HCMV - genome wide average | 0.22 | This Study |
| canine parvovirus | 0.29 |  |
| dengue virus Type III | 0.3 |  |
| hepatitis C Virus (min)^1^ | 0.37 |  |
| HCMV - maximal ORF value | 0.64 | This Study |
| hepatitis B virus (S Region)^2^ | 0.67 |  |
| hepatitis B virus (C Region)^2^ | 1.5 |  |
| human immunodeficiency virus-1 (max)^1^ | 2.5 |  |
| hepatitis C virus (max)^1^ | 4.1 |  |

1. In the studies for Hepatitis C virus and Human Immunodeficiency Virus, there was a range of values reported for intrahost nucleotide diversity. In this table are listed the minimum and maximum values from these studies.

2. Two regions of the Hepatitis B Virus genome were studied (S and C Region) and showed different average nucleotide diversity values. Both values are listed.

**Table S4 References**

1. Emmenegger EJ, Troyer RM, Kurath G (2003) Characterization of the mutant spectra of a fish RNA virus within individual hosts during natural infections. Virus Res 96: 15-25.

2. Jerzak G, Bernard KA, Kramer LD, Ebel GD (2005) Genetic variation in West Nile virus from naturally infected mosquitoes and birds suggests quasispecies structure and strong purifying selection. J Gen Virol 86: 2175-2183.

3. Salazar-Gonzalez JF, Salazar MG, Keele BF, Learn GH, Giorgi EE, et al. (2009) Genetic identity, biological phenotype, and evolutionary pathways of transmitted/founder viruses in acute and early HIV-1 infection. J Exp Med 206: 1273-1289.

4. Battilani M, Scagliarini A, Ciulli S, Morganti L, Prosperi S (2006) High genetic diversity of the VP2 gene of a canine parvovirus strain detected in a domestic cat. Virology 352: 22-26.

5. Holmes EC (2003) Patterns of Intra- and Interhost Nonsynonymous Variation Reveal Strong Purifying Selection in Dengue Virus. J Virol 77: 11296-11298.

6. Sakai A, Kaneko S, Honda M, Matsushita E, Kobayashi K (1999) Quasispecies of hepatitis C virus in serum and in three different parts of the liver of patients with chronic hepatitis. Hepatology 30: 556-561.

7. Boot HJ, Cremer J, Koedijk FD, van Ballegooijen WM, Op de Coul EL (2008) Improved tracing of hepatitis B virus transmission chains by phylogenetic analysis based on C region sequences. J Med Virol 80: 233-241.
