# Supplementary material for: Extensive Genome-Wide Variability of Human Cytomegalovirus in Congenitally Infected Infants
Source: PLoS Pathog. 2011 May 19;7(5):e1001344. doi: 10.1371/journal.ppat.1001344 (PMC3098220; doi:10.1371/journal.ppat.1001344)
Supplement: Table S5 — ORFs that overlap selective sweeps in the HCMV genome (0.06 MB PDF) [file ppat.1001344.s014.pdf]

Table S5: ORFs that overlap selective sweeps in the HCMV genome

	ORF[1]	Kinetics	Function	ORF Start	ORF End	Sweep Window Start	Sweep Window End	CLR[2]	p value[3] (<)	% of ORF in Sweep Window
U01	UL9	L	Glycoprotein	16947	17648	17386	17621	5.11	0.044	34
	UL10	unk	Unknown	17776	18540	17622	17857	5.66	0.019	41
	UL10	unk	Unknown	17776	18540	17857	18092	5.18	0.043	
	UL25	L	Matrix/Tegument	30402	32372	31285	31520	8.08	0.002	48
	UL25	L	Matrix/Tegument	30402	32372	31520	31755	9.83	0.001	
	UL25	L	Matrix/Tegument	30402	32372	31756	31991	9.96	0.001	
	UL25	L	Matrix/Tegument	30402	32372	31991	32226	7.06	0.004	
	UL27	E	Unknown	35008	33182	34347	34582	5.76	0.017	13
	UL45	L	Matrix/Tegument	60663	57943	58846	59081	6.50	0.006	60
	UL45	L	Matrix/Tegument	60663	57943	59082	59317	12.97	0.001	
	UL45	L	Matrix/Tegument	60663	57943	59317	59552	16.35	0.001	
	UL45	L	Matrix/Tegument	60663	57943	59553	59788	19.06	0.001	
	UL45	L	Matrix/Tegument	60663	57943	59788	60023	20.58	0.001	
	UL45	L	Matrix/Tegument	60663	57943	60024	60259	20.77	0.001	
	UL45	L	Matrix/Tegument	60663	57943	60260	60495	18.68	0.001	
	UL47	E-L	Matrix/Tegument	61678	60806	60731	60966	7.33	0.004	18
	UL80	L	capsid	116500	118626	117032	117267	5.59	0.023	11
	UL95	E-L	Matrix?	139697	133770	135170	135405	5.17	0.043	4
	UL100	E-L	Glycoprotein	147653	146538	146949	147184	5.91	0.011	42
	UL100	E-L	Glycoprotein	147653	146538	147184	147419	7.60	0.004	
	UL102	L	DNA Replication	147831	150452	147655	147890	10.35	0.001	92
	UL102	L	DNA Replication	147831	150452	147891	148126	15.41	0.001	
	UL102	L	DNA Replication	147831	150452	148127	148362	19.24	0.001	
	UL102	L	DNA Replication	147831	150452	148362	148597	21.93	0.001	
	UL102	L	DNA Replication	147831	150452	148598	148833	23.57	0.001	
	UL102	L	DNA Replication	147831	150452	148833	149068	24.27	0.001	
	UL102	L	DNA Replication	147831	150452	149069	149304	24.16	0.001	
	UL102	L	DNA Replication	147831	150452	149304	149539	23.17	0.001	
	UL102	L	DNA Replication	147831	150452	149540	149775	21.33	0.001	
	UL102	L	DNA Replication	147831	150452	149776	150011	18.69	0.001	
	UL102	L	DNA Replication	147831	150452	150011	150246	16.07	0.001	
	UL103	L	Matrix/Tegument	151364	150615	150482	150717	8.63	0.002	14
	UL136	unk	Unknown	188755	188033	187938	188173	5.60	0.023	52
	UL136	unk	Unknown	188755	188033	188173	188408	5.09	0.046	

	Feature	Kinetics	Function	ORF Start	ORF End	Sweep Window Start	Sweep Window End	CLR	p value (<)	% of ORF in Sweep Window
U04	UL9	L	Glycoprotein	16744	17445	16666	16901	6.37	0.041	22
	UL36	IE	Matrix/Tegument	49925	48595	48439	48674	15.15	0.001	94
	UL36	IE	Matrix/Tegument	49925	48595	48674	48909	18.40	0.001	
	UL36	IE	Matrix/Tegument	49925	48595	48909	49144	20.63	0.001	
	UL36	IE	Matrix/Tegument	49925	48595	49145	49380	20.72	0.001	
	UL36	IE	Matrix/Tegument	49925	48595	49380	49615	20.02	0.001	
	UL36	IE	Matrix/Tegument	49925	48595	49616	49851	18.10	0.001	
	UL47	E-L	Matrix/Tegument	61475	60603	60442	60677	6.99	0.018	9
	UL69	E-L	regulatory	101544	99316	99275	99510	7.06	0.018	9
	UL83	L	Matrix/Tegument	122138	120453	121398	121633	7.09	0.017	42
	UL83	L	Matrix/Tegument	122138	120453	121634	121869	11.81	0.001	
	UL83	L	Matrix/Tegument	122138	120453	121869	122104	17.30	0.001	
	UL84	E-L	DNA Replication	124173	122410	122340	122575	20.22	0.001	63
	UL84	E-L	DNA Replication	124173	122410	122575	122810	19.64	0.001	
	UL84	E-L	DNA Replication	124173	122410	122810	123045	17.79	0.001	
	UL84	E-L	DNA Replication	124173	122410	123046	123281	14.73	0.001	
	UL84	E-L	DNA Replication	124173	122410	123281	123516	9.56	0.002	
	UL104	E	capsid	153030	155900	152936	153171	6.34	0.044	5
	UL105	E	DNA Replication	153202	151109	152936	153171	6.34	0.044	11
	UL114	E	DNA Replication	164847	164095	163997	164232	6.29	0.048	18
	UL123	IE	regulatory	173887	172329	172941	173176	6.72	0.024	16
	UL145	unk	Unknown	181762	181370	181413	181648	7.13	0.017	60
	UL139	unk	Glycoprotein	186675	186259	186356	186591	6.57	0.03	56
	IRS1	IE	regulatory	195773	198316	196005	196240	7.58	0.009	18
	IRS1	IE	regulatory	195773	198316	196241	196476	7.25	0.016	
	US31	unk	Unknown	228815	229300	228719	228954	12.23	0.001	77
	US31	unk	Unknown	228815	229300	228955	229190	12.84	0.001	
	US32	L	Unknown	229418	229969	229190	229425	11.56	0.001	44
	US32	L	Unknown	229418	229969	229426	229661	7.39	0.012	

	Feature	Kinetics	Function	ORF Start	ORF End	Sweep Window Start	Sweep Window End	CLR	p value (<)	% of ORF in Sweep Window
U33	UL4	E	Glycoprotein	13615	14067	13770	14005	6.40	0.008	52
	UL150	unk	Unknown	194057	192138	192744	192979	6.21	0.011	12

1. ORFs can be listed multiple times if overlapping with multiple sweep windows.
2. CLR is the compositive likelihood ratio for the window as calculated by the program SweepFinder
3. Simulations under a standard neutral model were used to calculate p values for the data. Only significant results ($p < 0.05$) are shown.
