# Supplementary material for: Extensive Genome-Wide Variability of Human Cytomegalovirus in Congenitally Infected Infants
Source: PLoS Pathog. 2011 May 19;7(5):e1001344. doi: 10.1371/journal.ppat.1001344 (PMC3098220; doi:10.1371/journal.ppat.1001344)
Supplement: Table S6 — Whole genome interhost polymorphism data from patient samples (0.27 MB PDF) [file ppat.1001344.s015.pdf]

Table S6: Whole genome interhost polymorphism data from patient samples

			U01				U04				U33				Averages			
ORF	Kinetics	Function	Polymorhisms	Non-synonymous	Synonymous	dN/dS	Polymorhisms	Non-synonymous	Synonymous	dN/dS	Polymorhisms	Non-synonymous	Synonymous	dN/dS	Polymorhisms	Non-synonymous	Synonymous	dN/dS
RL1	unk	Regulatory	15	8	7	0.38	10	6	4	0.55	22	10	12	0.27	15.7	8.0	7.7	0.40
RL5A	E	Unknown	6	4	2	0.68	6	3	3	0.33	7	6	1	2.14	6.3	4.3	2.0	1.05
RL6	L	Unknown	3	2	1	0.69	4	2	2	0.33	3	2	1	1.04	3.3	2.0	1.3	0.69
RL10	E-L	Glycoprotein	9	1	8	0.04	10	5	5	0.33	5	1	4	0.03	8.0	2.3	5.7	0.13
RL11	L	Glycoprotein	10	3	7	0.12	20	3	17	0.03	15	7	8	0.28	15.0	4.3	10.7	0.14
RL12	E-L	Unknown	4	2	2	0.34	26	11	15	0.22	0	0	0	No Mutations	10.0	4.3	5.7	0.28
RL13	E-L	Unknown	4	2	2	0.34	12	7	5	1.20	0	0	0	No Mutations	5.3	3.0	2.3	0.77
UL1	E-L	Envelope	0	0	0	No Mutations	13	4	9	0.11	0	0	0	No Mutations	4.3	1.3	3.0	0.11
UL2	L	Unknown	8	4	4	0.30	2	2	0	Incalculable	0	0	0	No Mutations	3.3	2.0	1.3	0.30
UL4	E	Glycoprotein	10	6	4	0.54	20	7	12	0.13	4	3	1	2.44	11.3	5.3	5.7	1.04
UL5	E	Unknown	22	8	14	0.15	16	9	7	0.56	6	4	2	1.39	14.7	7.0	7.7	0.70
UL6	unk	Unknown	23	9	14	0.18	18	10	8	0.45	6	4	2	0.69	15.7	7.7	8.0	0.44
UL7	L	Unknown	25	12	12	0.34	21	3	18	0.01	10	4	6	0.20	18.7	6.3	12.0	0.19
UL8	unk	Unknown	13	6	7	0.26	10	7	3	1.40	8	5	3	0.64	10.3	6.0	4.3	0.77
UL9	L	Glycoprotein	6	3	3	0.34	1	0	1	0.00	8	3	5	0.18	5.0	2.0	3.0	0.17
UL10	unk	Unknown	32	14	18	0.22	28	13	15	0.26	12	4	8	0.11	24.0	10.3	13.7	0.20
UL11	E	Glycoprotein	18	6	12	0.12	11	4	7	0.15	24	11	13	0.26	17.7	7.0	10.7	0.18
UL13	E	Unknown	24	10	14	0.21	24	11	13	0.25	34	19	15	0.48	27.3	13.3	14.0	0.31
UL14	L	Unknown	7	0	7	0.00	15	4	11	0.09	15	1	14	0.00	12.3	1.7	10.7	0.03
UL15A	L	Unknown	4	2	2	0.33	2	1	1	0.34	2	0	2	0.00	2.7	1.0	1.7	0.22
UL16	E	Glycoprotein	16	4	12	0.07	5	2	3	0.22	13	2	11	0.05	11.3	2.7	8.7	0.11
UL17	E	Unknown	4	1	3	0.11	0	0	0	No Mutations	6	2	4	0.12	3.3	1.0	2.3	0.11
UL18	L	Glycoprotein	28	13	15	0.27	4	1	3	0.07	21	7	14	0.13	17.7	7.0	10.7	0.16
UL19	unk	Unknown	0	0	0	No Mutations	4	0	3	0.00	3	1	2	0.16	2.3	0.3	1.7	0.08
UL20	unk	Glycoprotein	30	12	18	0.19	26	8	17	0.08	27	8	18	0.07	27.7	9.3	17.7	0.11
UL21A	E-L	Glycoprotein	2	0	2	0.00	2	1	1	0.34	9	3	6	0.08	4.3	1.3	3.0	0.14
UL22A	unk	Glycoprotein	2	1	1	0.34	2	2	0	Incalculable	2	1	1	0.34	2.0	1.3	0.7	0.34
UL23	unk	Matrix/Tegument	8	4	4	0.34	2	0	2	0.00	9	5	4	0.45	6.3	3.0	3.3	0.26
UL24	E-L	Matrix/Tegument	9	2	7	0.08	0	0	0	No Mutations	9	2	7	0.07	6.0	1.3	4.7	0.07
UL25	L	Matrix/Tegument	9	2	7	0.08	1	0	0	0.00	30	10	18	0.14	13.3	4.0	8.3	0.07
UL26	E	Matrix/Tegument	2	0	2	0.00	6	3	3	0.34	2	1	1	0.34	3.3	1.3	2.0	0.23
UL27	E	Unknown	16	7	9	0.26	6	3	3	0.40	20	6	14	0.13	14.0	5.3	8.7	0.26
UL29	E	Unknown	23	1	22	0.01	4	1	3	0.09	30	9	21	0.12	19.0	3.7	15.3	0.07
UL30	unk	Unknown	2	1	1	0.34	0	0	0	No Mutations	8	4	4	0.33	3.3	1.7	1.7	0.34
UL31	L	Unknown	9	1	8	0.04	2	2	0	Incalculable	21	5	16	0.06	10.7	2.7	8.0	0.05
UL32	L	Matrix/Tegument	25	7	18	0.12	4	2	2	0.52	35	9	25	0.06	21.3	6.0	15.0	0.23
UL33	E	Glycoprotein	25	5	20	0.07	1	0	1	0.00	10	4	5	0.16	12.0	3.0	8.7	0.08
UL34	E-L	Regulatory	14	4	10	0.11	1	0	1	0.00	14	6	8	0.29	9.7	3.3	6.3	0.13
UL35	E	Matrix/Tegument	31	1	30	0.00	0	0	0	No Mutations	32	6	25	0.03	21.0	2.3	18.3	0.01
UL36	IE	Matrix/Tegument	25	7	18	0.11	2	1	1	0.34	23	19	4	2.33	16.7	9.0	7.7	0.93
UL37	IE	Regulatory	21	11	10	0.37	1	0	1	0.00	9	3	6	0.14	10.3	4.7	5.7	0.17
UL38	IE	Unknown	10	4	6	0.21	0	0	0	No Mutations	4	1	3	0.04	4.7	1.7	3.0	0.12
UL40	E-L	Regulatory	15	9	6	0.56	12	7	5	0.49	15	9	6	0.59	14.0	8.3	5.7	0.55
UL41A	L	Unknown	1	0	1	0.00	2	1	1	0.34	2	1	1	0.34	1.7	0.7	1.0	0.23
UL42	unk	Unknown	7	4	3	0.49	2	2	0	Incalculable	4	2	2	0.34	4.3	2.7	1.7	0.41
UL43	L	Matrix/Tegument	17	4	13	0.06	19	7	12	0.14	13	6	7	0.25	16.3	5.7	10.7	0.15
UL44	E-L	DNA Replication	20	2	18	0.03	16	6	9	0.16	17	5	12	0.09	17.7	4.3	13.0	0.09
UL45	L	Matrix/Tegument	37	6	31	0.05	17	4	13	0.08	40	11	29	0.07	31.3	7.0	24.3	0.07
UL47	E-L	Matrix/Tegument	7	0	7	0.00	5	0	5	0.00	7	2	5	0.09	6.3	0.7	5.7	0.03
UL46	E-L	capsid	42	7	35	0.05	31	5	26	0.03	36	9	26	0.07	36.3	7.0	29.0	0.05
UL48	L	capsid	97	13	84	0.04	99	32	65	0.10	80	25	47	0.11	92.0	23.3	65.3	0.08
UL48A	unk	capsid	6	0	6	0.00	9	3	6	0.10	4	2	2	0.33	6.3	1.7	4.7	0.14
UL49	E-L	capsid	23	7	16	0.12	13	3	10	0.06	11	4	6	0.27	15.7	4.7	10.7	0.15
UL50	unk	Nuclear Egress	10	4	6	0.22	6	3	3	0.34	14	3	10	0.04	10.0	3.3	6.3	0.20
UL51	unk	DNA Packaging	2	1	1	0.34	1	0	1	0.00	4	1	3	0.11	2.3	0.7	1.7	0.15
UL52	L	DNA Packaging	15	6	9	0.22	14	4	10	0.12	33	12	21	0.14	20.7	7.3	13.3	0.16
UL53	E	Nuclear Egress	17	8	9	0.29	9	2	7	0.04	17	7	10	0.21	14.3	5.7	8.7	0.18
UL54	E	DNA Replication	47	3	44	0.02	45	10	35	0.04	17	6	11	0.17	36.3	6.3	30.0	0.07
UL55	E	Glycoprotein	66	11	55	0.04	55	20	35	0.11	26	9	17	0.12	49.0	13.3	35.7	0.09
UL56	E	DNA Packaging	29	4	25	0.04	31	13	18	0.22	16	3	13	0.08	25.3	6.7	18.7	0.11
UL57	E	DNA Replication	17	0	17	0.00	30	7	23	0.07	17	4	12	0.03	21.3	3.7	17.3	0.03
UL69	E-L	Regulatory	18	6	12	0.14	10	2	7	0.12	10	4	6	0.19	12.7	4.0	8.3	0.15
UL70	E-L	DNA Replication	25	2	23	0.03	31	6	25	0.06	26	5	21	0.07	27.3	4.3	23.0	0.05
UL71	unk	Unknown	11	2	9	0.05	12	5	7	0.21	6	1	5	0.07	9.7	2.7	7.0	0.11

UL72	L	Unknown	8	0	8	0.00	12	5	7	0.20	11	2	9	0.03	10.3	2.3	8.0	0.08
UL73	E-L	Glycoprotein	20	6	14	0.09	13	1	12	0.00	13	2	11	0.04	15.3	3.0	12.3	0.04
UL74	unk	Glycoprotein	20	7	13	0.17	26	15	11	0.63	9	6	3	1.86	18.3	9.3	9.0	0.89
UL75	E-L	Glycoprotein	27	6	21	0.08	30	9	20	0.10	40	12	28	0.08	32.3	9.0	23.0	0.09
UL76	unk	Regulatory	12	3	9	0.07	17	6	10	0.12	12	5	7	0.21	13.7	4.7	8.7	0.13
UL77	unk	DNA Packaging	32	9	23	0.12	23	10	13	0.24	17	5	12	0.08	24.0	8.0	16.0	0.14
UL78	E	Regulatory	24	8	16	0.14	1	0	1	0.00	14	2	12	0.04	13.0	3.3	9.7	0.06
UL79	E-L	Unknown	9	2	7	0.04	2	1	1	0.34	6	1	5	0.01	5.7	1.3	4.3	0.13
UL80	L	capsid	32	10	22	0.13	4	1	3	0.11	25	7	18	0.08	20.3	6.0	14.3	0.11
UL80.5	unk	capsid	18	5	13	0.11	1	0	1	0.00	18	4	14	0.04	12.3	3.0	9.3	0.05
UL82	L	Matrix/Tegument	32	5	27	0.03	0	0	0	No Mutations	10	3	7	0.12	14.0	2.7	11.3	0.07
UL83	L	Matrix/Tegument	21	2	19	0.01	1	0	1	0.00	18	5	12	0.06	13.3	2.3	10.7	0.02
UL84	E-L	DNA Replication	23	7	16	0.10	0	0	0	No Mutations	22	6	15	0.07	15.0	4.3	10.3	0.09
UL85	E-L	capsid	9	0	9	0.00	0	0	0	No Mutations	6	1	5	0.01	5.0	0.3	4.7	0.01
UL86	E-L	capsid	96	8	88	0.02	3	0	2	0.00	26	4	21	0.05	41.7	4.0	37.0	0.02
UL87	E-L	Matrix?	50	18	32	0.17	12	6	6	0.34	16	6	10	0.16	26.0	10.0	16.0	0.22
UL88	unk	Matrix/Tegument	13	1	12	0.01	5	1	4	0.05	11	4	6	0.25	9.7	2.0	7.3	0.10
UL91	L	Unknown	9	2	7	0.10	10	3	7	0.15	5	3	2	0.52	8.0	2.7	5.3	0.25
UL92	L	Unknown	3	1	2	0.17	2	1	1	0.34	5	0	5	0.00	3.3	0.7	2.7	0.17
UL93	L	DNA Packaging	5	0	5	0.00	5	4	1	4.54	8	3	5	0.13	6.0	2.3	3.7	1.56
UL94	L	Matrix/Tegument	30	7	23	0.09	30	12	18	0.17	13	5	8	0.15	24.3	8.0	16.3	0.14
UL95	E-L	Matrix?	20	0	20	0.00	19	7	12	0.16	7	1	6	0.06	15.3	2.7	12.7	0.07
UL89	E-L	DNA Packaging	21	4	17	0.07	20	9	11	0.25	12	5	7	0.23	17.7	6.0	11.7	0.18
UL96	E-L	Matrix/Tegument	2	0	2	0.00	2	1	1	0.34	3	1	2	0.08	2.3	0.7	1.7	0.14
UL97	E-L	Matrix/Tegument	16	2	14	0.04	18	6	12	0.19	14	5	9	0.18	16.0	4.3	11.7	0.13
UL98	E-L	DNA Replication	13	2	11	0.04	12	1	11	0.00	8	1	7	0.03	11.0	1.3	9.7	0.02
UL99	L	Envelope	6	5	1	7.38	10	3	7	0.09	3	1	2	0.08	6.3	3.0	3.3	2.52
UL100	E-L	Glycoprotein	2	1	1	0.34	14	5	7	0.34	21	8	13	0.15	12.3	4.7	7.0	0.28
UL102	L	DNA Replication	2	0	2	0.00	29	6	23	0.04	32	6	26	0.04	21.0	4.0	17.0	0.03
UL103	L	Matrix/Tegument	0	0	0	No Mutations	6	2	4	0.13	10	2	8	0.02	5.3	1.3	4.0	0.07
UL105	E	DNA Replication	9	0	9	0.00	25	5	20	0.04	18	6	12	0.13	17.3	3.7	13.7	0.05
UL104	E	capsid	14	4	10	0.13	13	2	11	0.07	10	5	5	0.45	12.3	3.7	8.7	0.22
UL111A	E-L	Immune Evasion	0	0	0	No Mutations	5	2	3	0.19	0	0	0	No Mutations	1.7	0.7	1.0	0.19
UL112	E	DNA Replication	5	2	3	0.17	25	14	11	0.49	3	1	2	0.17	11.0	5.7	5.3	0.28
UL114	E	DNA Replication	5	0	5	0.00	8	2	6	0.07	2	0	2	0.00	5.0	0.7	4.3	0.02
UL115	L	Glycoprotein	8	3	5	0.19	7	2	5	0.13	0	0	0	No Mutations	5.0	1.7	3.3	0.16
UL116	E-L	Unknown	5	2	3	0.23	14	5	9	0.17	2	2	0	Incalculable	7.0	3.0	4.0	0.20
UL117	L	Unknown	4	0	4	0.00	11	3	8	0.05	18	5	13	0.08	11.0	2.7	8.3	0.04
UL119	E	Glycoprotein	15	3	12	0.08	22	13	9	0.71	22	15	7	1.49	19.7	10.3	9.3	0.76
UL120	L	Unknown	5	0	5	0.00	17	9	8	0.47	5	3	2	0.69	9.0	4.0	5.0	0.39
UL121	L	Unknown	19	9	10	0.28	19	7	12	0.15	13	7	6	0.43	17.0	7.7	9.3	0.29
UL122	IE,L	Regulatory	34	9	25	0.08	29	17	12	1.22	21	16	5	2.38	28.0	14.0	14.0	1.23
UL123	IE	Regulatory	50	16	34	0.11	17	8	8	0.43	28	18	9	1.45	31.7	14.0	17.0	0.66
UL124	E	Unknown	11	4	6	0.15	9	5	3	1.04	7	3	2	1.39	9.0	4.0	3.7	0.86
UL128	E	Glycoprotein	4	2	2	0.34	5	3	2	0.51	2	1	1	0.34	3.7	2.0	1.7	0.39
UL130	E-L	Glycoprotein	11	2	9	0.07	10	4	6	0.17	9	2	7	0.09	10.0	2.7	7.3	0.11
UL131A	L	Glycoprotein	0	0	0	No Mutations	4	0	4	0.00	7	1	6	0.05	3.7	0.3	3.3	0.03
UL132	E-L	Glycoprotein	14	10	4	1.13	14	6	6	0.36	16	12	4	1.61	14.7	9.3	4.7	1.03
UL148	unk	Unknown	10	3	7	0.12	12	3	8	0.09	12	3	9	0.06	11.3	3.0	8.0	0.09
UL147A	unk	Unknown	10	3	7	0.09	5	0	5	0.00	8	2	6	0.06	7.7	1.7	6.0	0.05
UL147	E-L	Regulatory	8	2	6	0.08	12	5	7	0.26	15	6	9	0.18	11.7	4.3	7.3	0.17
UL146	unk	Regulatory	0	0	0	No Mutations	0	0	0	No Mutations	0	0	0	No Mutations	0.0	0.0	0.0	--
UL145	unk	Unknown	10	3	7	0.11	6	3	3	0.33	10	3	7	0.11	8.7	3.0	5.7	0.18
UL144	unk	Glycoprotein	10	2	7	0.08	19	5	13	0.07	15	6	8	0.18	14.7	4.3	9.3	0.11
UL142	unk	Glycoprotein	26	12	14	0.26	23	13	10	0.51	10	4	6	0.19	19.7	9.7	10.0	0.32
UL141	unk	Glycoprotein	32	3	29	0.03	29	8	21	0.06	14	4	10	0.07	25.0	5.0	20.0	0.05
UL140	unk	Unknown	3	2	1	0.69	12	3	8	0.07	11	2	9	0.04	8.7	2.3	6.0	0.27
UL139	unk	Glycoprotein	2	0	2	0.00	4	1	3	0.04	9	4	3	0.53	5.0	1.7	2.7	0.19
UL138	unk	Regulatory	4	1	3	0.11	13	1	12	0.00	8	0	8	0.00	8.3	0.7	7.7	0.04
UL136	unk	Unknown	0	0	0	No Mutations	8	0	5	0.00	13	3	8	0.08	7.0	1.0	4.3	0.04
UL135	unk	Unknown	3	0	3	0.00	5	2	3	0.23	19	6	13	0.08	9.0	2.7	6.3	0.10
UL133	unk	Unknown	0	0	0	No Mutations	24	7	17	0.10	23	9	14	0.18	15.7	5.3	10.3	0.14
UL148A	unk	Unknown	8	1	7	0.02	8	0	8	0.00	3	0	3	0.00	6.3	0.3	6.0	0.01
UL148B	unk	Unknown	4	1	3	0.10	3	1	2	0.08	4	1	3	0.10	3.7	1.0	2.7	0.10
UL148C	unk	Unknown	7	0	7	0.00	2	1	1	0.34	0	0	0	No Mutations	3.0	0.3	2.7	0.17
UL148D	unk	Unknown	7	3	4	0.22	6	4	2	1.04	3	2	1	0.68	5.3	3.0	2.3	0.65
UL150	unk	Unknown	96	51	45	0.36	65	33	31	0.36	47	24	22	0.36	69.3	36.0	32.7	0.36
IRS1	IE	Regulatory	22	5	17	0.10	14	5	9	0.15	13	7	6	0.51	16.3	5.7	10.7	0.25
US1	unk	Unknown	6	2	4	0.16	2	2	0	Incalculable	4	2	2	0.34	4.0	2.0	2.0	0.25

US2	E	Immune Evasion	4	1	3	0.17	7	3	4	0.34	4	3	1	1.18	5.0	2.3	2.7	0.56
US3	IE	Immune Evasion	15	10	5	0.97	12	6	6	0.40	0	0	0	No Mutations	9.0	5.3	3.7	0.68
US6	E-L	Immune Evasion	8	5	3	0.76	9	3	6	0.14	1	0	1	0.00	6.0	2.7	3.3	0.30
US7	E-L	Immune Evasion	22	7	15	0.12	2	2	0	Incalculable	27	17	9	1.24	17.0	8.7	8.0	0.68
US8	E	Immune Evasion	5	4	1	2.44	10	2	7	0.04	5	1	4	0.08	6.7	2.3	4.0	0.85
US9	E	Immune Evasion	10	2	8	0.08	10	3	7	0.10	6	3	3	0.34	8.7	2.7	6.0	0.17
US10	E	Immune Evasion	15	1	14	0.02	16	6	9	0.15	14	6	8	0.21	15.0	4.3	10.3	0.13
US11	E	Immune Evasion	6	2	4	0.13	7	4	3	0.60	5	3	2	0.69	6.0	3.0	3.0	0.47
US12	E	Unknown	8	1	7	0.05	9	4	5	0.22	10	2	8	0.05	9.0	2.3	6.7	0.10
US13	E	Unknown	2	0	2	0.00	3	0	3	0.00	2	1	1	0.34	2.3	0.3	2.0	0.11
US14	E	Unknown	28	5	23	0.05	28	12	16	0.21	24	11	13	0.26	26.7	9.3	17.3	0.17
US15	E-L	Unknown	3	1	2	0.11	5	2	3	0.19	5	2	3	0.19	4.3	1.7	2.7	0.16
US16	E	Unknown	5	2	3	0.23	7	4	3	0.49	6	4	1	4.53	6.0	3.3	2.3	1.75
US17	E	Unknown	17	5	12	0.12	1	0	1	0.00	7	3	4	0.19	8.3	2.7	5.7	0.10
US18	E	Unknown	11	0	11	0.00	4	2	2	0.34	10	3	7	0.06	8.3	1.7	6.7	0.13
US19	E	Unknown	1	1	0	Incalculable	2	1	1	0.34	7	5	2	1.84	3.3	2.3	1.0	1.09
US20	E	Unknown	3	1	2	0.17	0	0	0	No Mutations	14	2	12	0.03	5.7	1.0	4.7	0.10
US21	unk	Unknown	9	0	9	0.00	5	3	2	0.69	9	5	4	0.51	7.7	2.7	5.0	0.40
US22	E	Matrix/Tegument	25	4	21	0.05	14	7	5	1.21	14	6	8	0.26	17.7	5.7	11.3	0.50
US23	E	Matrix/Tegument	29	3	26	0.01	40	10	29	0.06	40	6	34	0.01	36.3	6.3	29.7	0.03
US24	E	Matrix/Tegument	13	0	13	0.00	11	1	10	0.01	13	3	9	0.05	12.3	1.3	10.7	0.02
US26	E	Unknown	6	3	3	0.34	5	2	3	0.23	15	4	11	0.09	8.7	3.0	5.7	0.22
US27	E	Glycoprotein	11	5	6	0.26	1	1	0	Incalculable	12	7	5	0.72	8.0	4.3	3.7	0.49
US28	E	Envelope	13	3	10	0.10	1	0	1	0.00	19	6	13	0.13	11.0	3.0	8.0	0.08
US29	E-L	Unknown	15	5	10	0.15	2	0	2	0.00	20	6	14	0.10	12.3	3.7	8.7	0.08
US30	E	Unknown	24	10	14	0.21	0	0	0	No Mutations	16	2	14	0.04	13.3	4.0	9.3	0.13
US31	unk	Unknown	3	0	3	0.00	0	0	0	No Mutations	5	0	5	0.00	2.7	0.0	2.7	0.00
US32	L	Unknown	6	1	5	0.06	0	0	0	No Mutations	5	2	3	0.19	3.7	1.0	2.7	0.13
US34	E	Unknown	12	8	4	0.77	0	0	0	No Mutations	15	10	5	0.93	9.0	6.0	3.0	0.85
US34A	unk	Unknown	3	1	2	0.16	0	0	0	No Mutations	7	5	2	4.14	3.3	2.0	1.3	2.15
TRS1	IE	Regulatory	22	3	19	0.03	23	5	17	0.06	23	6	17	0.08	22.7	4.7	17.7	0.06
