# Supplementary material for: Extensive Genome-Wide Variability of Human Cytomegalovirus in Congenitally Infected Infants
Source: PLoS Pathog. 2011 May 19;7(5):e1001344. doi: 10.1371/journal.ppat.1001344 (PMC3098220; doi:10.1371/journal.ppat.1001344)
Supplement: Table S7 — Genome Wide McDonald-Kreitman Test (0.19 MB DOC) [file ppat.1001344.s016.doc]

**Table S7: Genome Wide McDonald-Kreitman Test**

| **ORF** | **Polymorphic Nonsynonymous/ Synonymous Ratio** | **Divergent Nonsynonymous/ Synonymous Ratio** | **Divergent/ Polymorphic Ratio** | **p values** | **(D_N_/D_S_)/(P_N_/P_S_) Ratio** |
| --- | --- | --- | --- | --- | --- |
| **RL13** | 2.64 | 0.57 | 0.22 | 0.011 | Significantly less than 1 |
| **UL6** | 4.89 | 2.32 | 0.47 | 0.049 | Significantly less than 1 |
| **UL8** | 2.50 | 0.83 | 0.33 | 0.031 | Significantly less than 1 |
| **UL22A** | 2.76 | 0.71 | 0.26 | <0.001 | Significantly less than 1 |
| **UL55** | 6.86 | 3.46 | 0.50 | 0.021 | Significantly less than 1 |
| **UL74** | 23.00 | 3.03 | 0.13 | 0.001 | Significantly less than 1 |
| **UL100** | 6.50 | 1.67 | 0.26 | 0.001 | Significantly less than 1 |
| **UL124** | 10.00 | 2.18 | 0.22 | 0.030 | Significantly less than 1 |
| **US6** | Incalculable | 2.73 | Incalculable | 0.048 | Significantly less than 1 |
| **US8** | 2.33 | 0.52 | 0.22 | 0.019 | Significantly less than 1 |
| **US29** | 19.00 | 3.32 | 0.17 | 0.029 | Significantly less than 1 |
| **RL1** | 0.60 | 0.91 | 1.52 | 0.340 | Not significant |
| **RL11** | 1.07 | 1.08 | 1.01 | 0.983 | Not significant |
| **RL12** | 2.16 | 2.22 | 1.03 | 0.877 | Not significant |
| **UL2** | 0.50 | 0.59 | 1.18 | 0.152 | Not significant |
| **UL4** | 1.18 | 1.78 | 1.51 | 0.244 | Not significant |
| **UL5** | 3.40 | 2.22 | 0.65 | 0.420 | Not significant |
| **UL7** | 1.43 | 0.96 | 0.67 | 0.194 | Not significant |
| **UL9** | 5.33 | 2.58 | 0.48 | 0.293 | Not significant |
| **UL10** | 3.89 | 2.12 | 0.55 | 0.115 | Not significant |
| **UL11** | 2.00 | 1.83 | 0.91 | 0.794 | Not significant |
| **UL13** | 2.89 | 2.48 | 0.86 | 0.699 | Not significant |
| **UL15A** | 4.00 | 1.40 | 0.35 | 0.319 | Not significant |
| **UL16** | 1.43 | 1.94 | 1.36 | 0.599 | Not significant |
| **UL17** | 1.00 | 1.54 | 1.54 | 0.682 | Not significant |
| **UL18** | 4.00 | 4.10 | 1.03 | 0.533 | Not significant |
| **UL19** | 0.67 | 0.88 | 1.32 | 1.000 | Not significant |
| **UL20** | 2.06 | 2.04 | 0.99 | 1.000 | Not significant |
| **UL23** | 0.71 | 0.70 | 0.98 | 0.977 | Not significant |
| **UL26** | 0.63 | 1.35 | 2.16 | 0.105 | Not significant |
| **UL27** | 0.72 | 0.62 | 0.86 | 0.691 | Not significant |
| **UL30** | 1.50 | 2.68 | 1.79 | 0.616 | Not significant |
| **UL31** | 10.00 | 4.55 | 0.46 | 0.444 | Not significant |
| **UL34** | 0.63 | 1.24 | 1.99 | 0.263 | Not significant |
| **UL36** | 2.09 | 2.34 | 1.12 | 0.770 | Not significant |
| **UL37** | 0.91 | 0.76 | 0.84 | 0.585 | Not significant |
| **UL38** | 2.20 | 0.97 | 0.44 | 0.107 | Not significant |
| **UL40** | 0.85 | 1.59 | 1.88 | 0.106 | Not significant |
| **UL42** | 0.80 | 0.76 | 0.95 | 0.937 | Not significant |
| **UL45** | 3.13 | 3.32 | 1.06 | 0.878 | Not significant |
| **UL48** | 3.33 | 3.72 | 1.12 | 0.882 | Not significant |
| **UL49** | 2.33 | 2.79 | 1.19 | 0.670 | Not significant |
| **UL50** | 0.75 | 0.94 | 1.26 | 0.677 | Not significant |
| **UL51** | 20.00 | 1.06 | 0.05 | 0.172 | Not significant |
| **UL52** | 4.14 | 2.36 | 0.57 | 0.187 | Not significant |
| **UL53** | 2.00 | 2.21 | 1.10 | 0.207 | Not significant |
| **UL69** | 1.67 | 2.32 | 1.39 | 0.439 | Not significant |
| **UL73** | 1.38 | 1.61 | 1.16 | 0.707 | Not significant |
| **UL75** | 2.42 | 2.14 | 0.89 | 0.669 | Not significant |
| **UL76** | 1.80 | 1.77 | 0.98 | 0.963 | Not significant |
| **UL77** | 0.47 | 0.33 | 0.69 | 0.249 | Not significant |
| **UL78** | 5.00 | 4.26 | 0.85 | 0.815 | Not significant |
| **UL79** | 0.22 | 0.52 | 2.34 | 0.163 | Not significant |
| **UL80** | 1.37 | 1.43 | 1.05 | 0.883 | Not significant |
| **UL80.5** | 1.80 | 1.12 | 0.62 | 0.242 | Not significant |
| **UL84** | 2.00 | 2.68 | 1.34 | 0.683 | Not significant |
| **UL85** | 0.00 | 0.65 | Incalculable | 0.055 | Not significant |
| **UL87** | 1.67 | 1.03 | 0.62 | 0.197 | Not significant |
| **UL89** | 3.25 | 1.33 | 0.41 | 0.122 | Not significant |
| **UL92** | 0.20 | 0.10 | 0.52 | 0.605 | Not significant |
| **UL94** | 2.31 | 2.98 | 1.29 | 0.468 | Not significant |
| **UL95** | 7.67 | 3.35 | 0.44 | 0.175 | Not significant |
| **UL96** | 0.67 | 1.15 | 1.72 | 0.665 | Not significant |
| **UL97** | 2.00 | 2.57 | 1.28 | 0.348 | Not significant |
| **UL98** | 0.47 | 0.78 | 1.66 | 0.031 | Not significant |
| **UL99** | 0.80 | 1.58 | 1.98 | 0.312 | Not significant |
| **UL102** | 0.51 | 0.72 | 1.40 | 0.262 | Not significant |
| **UL104** | 1.47 | 1.14 | 0.78 | 0.472 | Not significant |
| **UL112** | 1.30 | 1.27 | 0.97 | 0.953 | Not significant |
| **UL115** | 5.50 | 2.61 | 0.47 | 0.331 | Not significant |
| **UL116** | 3.40 | 2.68 | 0.79 | 0.646 | Not significant |
| **UL117** | 1.71 | 2.25 | 1.31 | 0.579 | Not significant |
| **UL119** | 2.00 | 1.60 | 0.80 | 0.464 | Not significant |
| **UL121** | 3.00 | 4.00 | 1.33 | 0.631 | Not significant |
| **UL122** | 0.96 | 1.05 | 1.09 | 0.050 | Not significant |
| **UL123** | 2.00 | 1.91 | 0.96 | 0.904 | Not significant |
| **UL128** | 1.88 | 3.47 | 1.85 | 0.267 | Not significant |
| **UL131A** | 1.78 | 1.07 | 0.60 | 0.098 | Not significant |
| **UL132** | 1.00 | 1.66 | 1.66 | 0.074 | Not significant |
| **UL133** | 2.33 | 2.48 | 1.06 | 0.885 | Not significant |
| **UL135** | 4.60 | 2.49 | 0.54 | 0.159 | Not significant |
| **UL136** | 1.83 | 2.01 | 1.10 | 0.860 | Not significant |
| **UL138** | 1.43 | 2.48 | 1.74 | 0.281 | Not significant |
| **UL139** | 1.20 | 1.25 | 1.04 | 0.945 | Not significant |
| **UL140** | 2.40 | 2.39 | 1.00 | 0.994 | Not significant |
| **UL141** | 1.71 | 2.08 | 1.21 | 0.589 | Not significant |
| **UL142** | 1.42 | 1.06 | 0.74 | 0.335 | Not significant |
| **UL144** | 1.11 | 0.94 | 0.84 | 0.721 | Not significant |
| **UL146** | Incalculable | 4.11 | Incalculable | 0.316 | Not significant |
| **UL147** | 0.88 | 1.24 | 1.42 | 0.584 | Not significant |
| **UL148A** | 1.50 | 1.08 | 0.72 | 0.560 | Not significant |
| **UL148B** | 0.00 | 1.16 | Incalculable | 0.110 | Not significant |
| **UL148D** | 1.00 | 0.94 | 0.94 | 0.955 | Not significant |
| **UL150** | 2.15 | 1.78 | 0.83 | 0.356 | Not significant |
| **IRS1** | 1.15 | 1.32 | 1.14 | 0.557 | Not significant |
| **US1** | 1.00 | 0.52 | 0.52 | 0.645 | Not significant |
| **US2** | 0.50 | 0.57 | 1.13 | 0.886 | Not significant |
| **US3** | 1.00 | 1.58 | 1.58 | 0.405 | Not significant |
| **US7** | 1.44 | 2.10 | 1.46 | 0.188 | Not significant |
| **US9** | 0.60 | 1.09 | 1.82 | 0.111 | Not significant |
| **US16** | 9.00 | 4.03 | 0.45 | 0.437 | Not significant |
| **US17** | 1.00 | 1.35 | 1.35 | 0.640 | Not significant |
| **US19** | 1.33 | 1.83 | 1.38 | 0.701 | Not significant |
| **US20** | 1.13 | 1.07 | 0.95 | 1.000 | Not significant |
| **US21** | 0.08 | 0.20 | 2.62 | 0.342 | Not significant |
| **US24** | 0.08 | 0.28 | 3.39 | 0.216 | Not significant |
| **US26** | 0.08 | 0.74 | 8.87 | 0.037 | Not significant |
| **US27** | 1.57 | 1.49 | 0.95 | 0.909 | Not significant |
| **US28** | 0.71 | 0.81 | 1.15 | 0.845 | Not significant |
| **US30** | 5.20 | 2.42 | 0.47 | 0.082 | Not significant |
| **US31** | 0.00 | 0.39 | Incalculable | 0.215 | Not significant |
| **US32** | 0.40 | 0.56 | 1.40 | 0.688 | Not significant |
| **US34** | 1.78 | 1.63 | 0.92 | 0.520 | Not significant |
| **US34A** | 7.00 | 3.40 | 0.49 | 0.671 | Not significant |
| **TRS1** | 1.67 | 1.94 | 1.16 | 0.393 | Not significant |
| **RL10** | 0.36 | 1.13 | 3.17 | 0.044 | Significantly greater than 1 |
| **UL14** | 0.00 | 1.01 | Incalculable | <0.001 | Significantly greater than 1 |
| **UL21A** | 0.00 | 0.51 | Incalculable | 0.034 | Significantly greater than 1 |
| **UL24** | 0.27 | 0.85 | 3.12 | 0.047 | Significantly greater than 1 |
| **UL25** | 1.05 | 1.88 | 1.80 | 0.043 | Significantly greater than 1 |
| **UL29** | 0.04 | 0.53 | 12.15 | 0.002 | Significantly greater than 1 |
| **UL32** | 0.67 | 1.55 | 2.33 | 0.022 | Significantly greater than 1 |
| **UL33** | 0.48 | 1.06 | 2.21 | 0.030 | Significantly greater than 1 |
| **UL35** | 0.16 | 0.94 | 5.78 | <0.001 | Significantly greater than 1 |
| **UL41A** | 0.00 | 1.11 | Incalculable | 0.041 | Significantly greater than 1 |
| **UL43** | 0.24 | 0.87 | 3.71 | 0.013 | Significantly greater than 1 |
| **UL44** | 0.08 | 0.36 | 4.62 | 0.014 | Significantly greater than 1 |
| **UL46** | 0.33 | 1.67 | 5.00 | 0.011 | Significantly greater than 1 |
| **UL47** | 0.10 | 0.29 | 2.90 | 0.022 | Significantly greater than 1 |
| **UL48** | 1.56 | 2.27 | 1.45 | 0.037 | Significantly greater than 1 |
| **UL54** | 0.25 | 0.58 | 2.28 | 0.008 | Significantly greater than 1 |
| **UL56** | 0.31 | 0.55 | 1.76 | 0.046 | Significantly greater than 1 |
| **UL57** | 0.25 | 0.86 | 3.43 | 0.003 | Significantly greater than 1 |
| **UL70** | 0.22 | 0.53 | 2.46 | 0.009 | Significantly greater than 1 |
| **UL71** | 1.00 | 2.38 | 2.38 | 0.029 | Significantly greater than 1 |
| **UL72** | 0.15 | 0.52 | 3.47 | 0.036 | Significantly greater than 1 |
| **UL82** | 0.64 | 1.50 | 2.35 | 0.013 | Significantly greater than 1 |
| **UL83** | 0.14 | 0.94 | 6.58 | 0.044 | Significantly greater than 1 |
| **UL86** | 0.07 | 0.28 | 4.13 | 0.001 | Significantly greater than 1 |
| **UL88** | 0.23 | 0.86 | 3.72 | 0.030 | Significantly greater than 1 |
| **UL91** | 0.29 | 1.42 | 4.96 | 0.034 | Significantly greater than 1 |
| **UL93** | 0.41 | 0.97 | 2.36 | 0.030 | Significantly greater than 1 |
| **UL103** | 0.08 | 1.26 | 15.12 | 0.001 | Significantly greater than 1 |
| **UL105** | 0.21 | 0.72 | 3.36 | 0.045 | Significantly greater than 1 |
| **UL114** | 0.01 | 0.75 | 75.02 | 0.007 | Significantly greater than 1 |
| **UL120** | 0.54 | 1.94 | 3.60 | 0.015 | Significantly greater than 1 |
| **UL130** | 0.45 | 1.22 | 2.69 | 0.042 | Significantly greater than 1 |
| **UL145** | 0.23 | 0.67 | 2.91 | 0.035 | Significantly greater than 1 |
| **UL147A** | 0.40 | 1.03 | 2.59 | 0.044 | Significantly greater than 1 |
| **UL148** | 0.33 | 1.17 | 3.52 | 0.012 | Significantly greater than 1 |
| **UL148C** | 0.00 | 1.05 | Incalculable | 0.025 | Significantly greater than 1 |
| **US10** | 0.13 | 1.39 | 11.12 | 0.000 | Significantly greater than 1 |
| **US11** | 0.50 | 1.97 | 3.94 | 0.019 | Significantly greater than 1 |
| **US12** | 0.07 | 0.69 | 9.59 | 0.008 | Significantly greater than 1 |
| **US13** | 0.00 | 0.99 | Incalculable | 0.034 | Significantly greater than 1 |
| **US14** | 0.30 | 0.81 | 2.71 | 0.009 | Significantly greater than 1 |
| **US15** | 0.20 | 1.34 | 6.68 | 0.047 | Significantly greater than 1 |
| **US18** | 0.00 | 0.92 | Incalculable | 0.000 | Significantly greater than 1 |
| **US22** | 0.17 | 0.48 | 2.87 | 0.025 | Significantly greater than 1 |
| **US23** | 0.40 | 0.95 | 2.38 | 0.013 | Significantly greater than 1 |
