# Supplementary material for: Extensive Genome-Wide Variability of Human Cytomegalovirus in Congenitally Infected Infants
Source: PLoS Pathog. 2011 May 19;7(5):e1001344. doi: 10.1371/journal.ppat.1001344 (PMC3098220; doi:10.1371/journal.ppat.1001344)
Supplement: Table S8 — Primers used in this study to amplify HCMV Genome (0.15 MB DOC) [file ppat.1001344.s017.doc]

| **Table S8: Primers used in this study to amplify HCMV Genome** | | |  |
| --- | --- | --- | --- |
| **Primer^1^** | **Sequence** | **Sequence Length** | **Notes** |
| 1A | GGGGTGTTTTTAGCGGGGGG | 20 |  |
| 1B | CAGCGCGCCGAAACACCGTCC | 21 |  |
| 2A | TGCGTGTCGTCCCACGGTATTT | 22 |  |
| 2B | ATCGGGTGGGAAACATGGGAT | 21 |  |
| 3A | TGCTTCATTCGGACTGATTTTCTT | 24 |  |
| 3B | ATTGTACAGCGTCAGATTTTGGC | 23 |  |
| 4A | ACCCCTAAAGCTCGCCAAC | 19 |  |
| 4B | CGTTGATTACCTCGTAACTGCGG | 23 |  |
| 5A | CATTATGAGTACCCCACGCAT | 21 |  |
| 5A-REV | GTAGCCTAGAAACATGTTAGCG | 22 |  |
| 5B-FORW | CGCTAACATGTTTCTAGGCTAC | 22 |  |
| 5B | TATTTGTACGCGTTGTATTGGC | 22 |  |
| 6A | ACAACTACGTGCCGTCGCTG | 20 |  |
| 6A-REV | GAAACACCGTTGGTTGGAGAAG | 22 |  |
| 6B-FORW | CTTCTCCAACCAACGGTGTTTC | 22 |  |
| 6B | GCTTCATGGCTCTCCTCGTTTACTC | 25 |  |
| 7A | ATCGTACCGGCGTCTGAACAC | 21 |  |
| 7A-REV | GGAAGTAGCGGATTGCATTG | 20 |  |
| 7B-FORW | CAATGCAATCCGCTACTTCC | 20 |  |
| 7B | TCTTCGTCTCCGTCGGTGGTGG | 22 |  |
| 8A | CTTTGGCGGCACCTTCTCAG | 20 |  |
| 8A-REV | GACGCGACGACGATCGTTTC | 20 |  |
| 8B-FORW | GAAACGATCGTCGTCGCGTC | 20 |  |
| 8B | TTTCGCAAACACGTCCACGATCT | 23 |  |
| 9A | CCGAAAACGGCGTACATGAAG | 21 |  |
| 9B | GCATGGTTTCCTCGTCGTAA | 20 |  |
| 10A | TGCTGCAACCGCTCATCACC | 20 |  |
| 10B | AGATCAGGGGGACGTGTACC | 20 |  |
| 11A | CCGCGTCCAACGACACATCCAC | 22 |  |
| 11A-REV | GATACGTCTCGGTGGTGATC | 20 |  |
| 11B-FORW | GATCACCACCGAGACGTATC | 20 |  |
| 11B | ATGTGCCCGCGATTTCTCAAG | 21 |  |
| 12A | GCTCGGAGGAGAGCCGAACG | 20 |  |
| 12A-R | CAAGACGACGTGAGACCCAC | 20 |  |
| 12B-F | GTGGGTCTCACGTCGTCTTG | 20 |  |
| 12B | GTCAGAGCATCAACGACGAG | 20 |  |
| 13A | CAGGTCCTTGCGAGCGTCG | 19 |  |
| 13A-REV | CTGCATCTACCCGACGTGG | 19 |  |
| 13B-FORW | CCACGTCGGGTAGATGCAG | 19 |  |
| 13B | TCTGTACCGTGGCGTGGCTTAG | 22 |  |
| 14A | TACCGTGAGCCAATTGGAGA | 20 |  |
| 14B | TTTGGTAGCCGTCCAGTAGC | 20 |  |
| 15A | CACGGTAACTTCTGTGGAGGA | 21 |  |
| 15B | TACGGAGTGCTGACGAAGTG | 20 |  |
| 16A | GACGCCGAATTGGATTTGC | 19 |  |
| 16A-REV | GATTGATGAAGGCCACCATGG | 21 |  |
| 16B-FORW | CCATGGTGGCCTTCATCAATC | 21 |  |
| 16B | TGGAGCAGGGTTTTCCCGTGT | 21 |  |
| 16B | TGGAGCAGGGTTTTCCCGTGT | 21 |  |
| 17A | AGGTGCCGATCGTAAAGAGA | 20 |  |
| 17A-REV | GTACCCCTATCGCGTGTGTTC | 21 |  |
| 17B-FORW | GAACACACGCGATAGGGGTAC | 21 |  |
| 17B | TTATTTGTGCACCGACTCCA | 20 |  |
| 18A | ATGGCGTATTCGTTGCATTT | 20 |  |
| 18A-REV | GTACACGTCATGGTGTCAATC | 21 |  |
| 18B-FORW | GATTGACACCATGACGTGTAC | 21 |  |
| 18B | CACGCCGGGTTTTATAGGTT | 20 |  |
| 19A | GCATGCGCCGGTAAAATTCCA | 21 |  |
| 19A-REV | CATGACGAGCGAGAGAACCGT | 21 |  |
| 19B-FORW | CCGCACCCCAACCGGCGCCA | 20 |  |
| 19B | GAGCTCATGCACACCGACTA | 20 |  |
| 20A | CCGTGCAGGTGGTGGTACTTC | 21 |  |
| 20B | TTCTGGAAGCAGCAATGTCGTAG | 23 |  |
| 21A | CGGTCGGTCAACATCGTAAG | 20 |  |
| 21A-REV | CACCTGCCTCTCACAAACACC | 21 |  |
| 21B-FORW | GGTGTTTGTGAGAGGCAGGTG | 21 |  |
| 21B | CAACAGTTCGTAAATATCGTTGACGTT | 27 |  |
| 22A | GTCTTCGCTCCGCGCACGGGCA | 22 |  |
| 22A-REV | GGGGGCGCCGTAGGACGCGGG | 21 |  |
| 22B-FORW | CGTCGCTTTCTCCCTCGGAG | 20 |  |
| 22B | CACGCGCTTAGGACGACTCTAT | 22 |  |
| 23A | TCGTGCGGAGATTTGTTCTC | 20 |  |
| 23A-REV | CGAACGRCGAGTCTCAAAC | 19 |  |
| 23B-FORW | GTTTGAGACTCGYCGTTCG | 19 |  |
| 23B | CGACGACGAATGGACGTTAC | 20 |  |
| 24A | GTAACGTCCATTCGTCGTCG | 20 |  |
| 24A-REV | CACCATCAGGTAAGGATAGTC | 21 |  |
| 24B-FORW | GACTATCCTTACCTGATGGTG | 21 |  |
| 24B | GCGGAATATTACTCATTCGCAAA | 23 |  |
| 25A | TTTGCGAATGAGTAATATTCCGC | 23 |  |
| 25A-REV | CAGACCAGTCTCAGGTGTAG | 20 |  |
| 25B-FORW | CTACACCTGAGACTGGTCTG | 20 |  |
| 25B | ACGACACCACTAGGGACGAC | 20 |  |
| 26A | GCATCATCTGGTAGCGCTTG | 20 |  |
| 26A-REV | GCTCATGTCCACGGCATAAC | 20 |  |
| 26B-FORW | GTTATGCCGTGGACATGAGC | 20 |  |
| 26B | GGGTGGACGTTGTGAAATCT | 20 |  |
| 27A | CGGACGACCTAGACGAAGAG | 20 |  |
| 27B | TCTACGTGGATTGGTGCGTA | 20 |  |
| 28A | AGCAGCAGCGCCAGGTTAG | 19 |  |
| 28A-REV | GGGATACCGAACTCCATGTAC | 21 |  |
| 28B-FORW | TCGCCGACAACTGGGTCATGT | 21 |  |
| 28B | CACTTAGGATCGATTTCGTGCG | 22 |  |
| 28B-FORW | TCGCCGACAACTGGGTCATGT | 21 |  |
| 29A | CGGTCTTCTTCTCCGATGAC | 20 |  |
| 29A-REV | CCACACAACCACCAGCGCAC | 20 |  |
| 29B-FORW | GTGCGCTGGTGGTTGTGTGG | 20 |  |
| 29B | CGCCAGTCGTCTGTCTCATTC | 21 |  |
| 30A | GCTCAGGTCTTCGATCCCTA | 20 |  |
| 30A-REV | GACAACGGTTACGCGAGTTTG | 21 |  |
| 30B-FORW | CAAACTCGCGTAACCGTTGTC | 21 |  |
| 30B | CAAGCGGCCTCTGATAACC | 19 |  |
| 31A | ATGCAGATCTCCTCAATGCG | 20 |  |
| 31A-REV | GATGTGTGCCGCGCTAAAATG | 21 |  |
| 31B-FORW | CATTTTAGCGCGGCACACATC | 21 |  |
| 31B | TTACATGAACTGGACGCTGCG | 21 |  |
| 32A | GGAATGGATGTCGGGCGTC | 19 |  |
| 32B | ATGGGGTCACCGCGTTGTTC | 20 |  |
| 33A | CTCGCATTTCGTCTTTCGGA | 20 |  |
| 33A-REV | CGTTCAGTGTTGGATCAGAC | 20 |  |
| 33B-FORW | GTCTGATCCAACACTGAACG | 20 |  |
| 33B | CGACGAGTCGTCTGCGTCCTC | 21 |  |
| 34A | CCAAATGCAGAGTGAGATGG | 20 |  |
| 34A-REV | CGTGGTAATCCAGGCCTATG | 20 |  |
| 34B-FORW | CATAGGCCTGGATTACCACG | 20 |  |
| 34B | TGCATCGCTTCGAGGTTGTC | 20 |  |
| 34B | CCGCGCTCTTCGTGTATTTC | 20 |  |
| 35A | CGAGAACCGGTCGCTCATAG | 20 |  |
| 35B | CGACAGCGATCTAACACGAA | 20 |  |
| 36A | CACCAGAGTAGGCCGTTCTTGGA | 23 |  |
| 36B | GCGTTGAGTCCCGAACAAACC | 21 |  |
| 37A | GGTCTGTAAACCACCCGAAA | 20 |  |
| 37B | GAGCCGGTAGCTCCTTCTTT | 20 |  |
| 38A | AGCTCGTTCCCATGGAAGCC | 20 |  |
| 38B | GGAAGACTAYCTGCAAGACGC | 21 |  |
| 38A-REV | GTACATCATGAGTTGCCTCATG | 22 |  |
| 38B-FORW | CATGAGGCAACTCATGATGTAC | 22 |  |
| 38B | TGGCTTTTTTGCTACGTCCGG | 21 |  |
| 39A | GACTGGCGATTTACGGTTATGTGGAC | 26 |  |
| 39B | GCCCATACAGCATGAATTCCCAG | 23 |  |
| 40A | AGCCAACCCTCTGCTTTTGC | 20 |  |
| 40B | CGTAACAGGATACGTTGGTGGC | 22 |  |
| 41A | CGTCTTCTTTTCGCCGTGCG | 20 |  |
| 41B | GGCTTTTATAGGCAGCGACGTG | 22 |  |
| 42A | CGGAGCTGTCGCACTTTCT | 19 |  |
| 42B | GGCTTTGTGGTCGTCAACTG | 20 |  |
| 43A | TTCTGTGGGTCGGGTGTAGCG | 21 | Use with 32A |
| 44A | ATCGGGCGCCAGAGCTAG | 18 |  |
| 44B | GAATCATCATGTGCCGCCGG | 20 |  |
| 45A | ACTGGAGTCCGTTCATCAGC | 20 |  |
| 45B | CGCTGTAGGGATAAATAGTGCG | 22 |  |
| 46A | TCACACTCTATCTCTTCACAGCGACA | 26 |  |
| 46B | GAACCGTTAGAATCAGGTCGC | 21 |  |
| 49A | ATGCTGTGGCGTATGTTCTCTC | 22 | Use with 41B |

**1**. Primer pairs for full length amplicons (~6 kb) are defined by A/B naming (e.g. primers 1A and 1B form a pair). When using shorter amplicons (~3 kb), pairs are defined by A/A-REV and B-FORW/B naming (e.g. 5A/5A-REV form a pair and 5B-FORW/5B form a pair).
