# Supplementary material for: Extensive Genome-Wide Variability of Human Cytomegalovirus in Congenitally Infected Infants
Source: PLoS Pathog. 2011 May 19;7(5):e1001344. doi: 10.1371/journal.ppat.1001344 (PMC3098220; doi:10.1371/journal.ppat.1001344)
Supplement: Table S9 — Assay for Quantitative Capabilities of High Throughput Sequencing Methodology (0.05 MB DOC) [file ppat.1001344.s018.doc]

**Table S9**: Assay for Quantitative Capabilities of High Throughput Sequencing Methodology

|  | **10% Mix^1^** | **1% Mix^1^** |
| --- | --- | --- |
| **Total Variants^2^** | 118 | 118 |
| **Detected** | 57 | 45 |
| **Undetected** | 61 | 73 |
| **Detection Rate (%)** | 48.3 | 38.1 |
| **Mean Frequency (%) ± S.E.M.^2^** | 9.9 ± 1.2 | 2.1 ± 0.25 |
| **Maximum Frequency (%)** | 40.1 | 8.6 |
| **Minimum Frequency (%)** | 2.5 | 1.9 |
| **Average Depth** | 697 | 901 |

1. 10% Mix is a PCR template mix that was 90% Toledo DNA and 10% AD169 DNA. 1% Mix was a template of 99% Toledo DNA and 1% AD169 DNA.

2. Variants are AD169 sequence detected using a Toledo reference genome. The mean frequency is the frequency at which AD169 variants were present in the sequenced population.
