# Supplementary material for: Extensive Genome-Wide Variability of Human Cytomegalovirus in Congenitally Infected Infants
Source: PLoS Pathog. 2011 May 19;7(5):e1001344. doi: 10.1371/journal.ppat.1001344 (PMC3098220; doi:10.1371/journal.ppat.1001344)
Supplement: Text S1 — Supporting Figure Legends and References (0.16 MB DOC) [file ppat.1001344.s019.doc]

**Text S1. Supporting Information**

**Supporting Information Figure Legends**

**Figure S1: Coverage of HCMV genome in previous sequencing studies:** The HCMV genome is depicted as a grey bar, with the subdivisions of the genome shown above as black bars. The coverage of the genome from previous sequencing studies is depicted with blue bars, with each blue bar representing a sequence study and the width of the bar being proportional to the length of the sequenced region. Although some regions have been sequenced in multiple studies (for example, *UL55* (gB)), for the purposes of this figure, we show the data from the study that sequenced the largest region. The data used to construct this figure are from , which are listed in Text S1.

**Figure S2: Single nucleotide variant counts and frequencies as a result of filtering:** Variants were called from BAC resequencing or clinical sequencing alignments normalized to an average depth of 250.  **A.** Variants of all frequencies called from the two datasets without filtering. **B.** Variants of all frequencies called from the two datasets after filtering with the variant caller algorithm. **C.** Same as **A** except that only variant frequencies from 0 - 0.2 are displayed. **D.** Same as **B** except that only variant frequencies from 0 - 0.2 are displayed.

**Figure S3: Coverage map of HCMV genomes sequenced directly from urine samples:** HCMV was sequenced from urine samples U01, U04 and U33 and the predicted coverage of the genomes was calculated. The black bars show a one dimensional representation of the genome, while the blue curve above is indicative of both the coverage and quality of sequence data across the genome. The presence of a blue curve indicates coverage of a base and the height of the blue curve is proportional to the quality. The major divisions of the HCMV genome are shown below the graph.

**Figure S4: Flowchart of genome type calling and detection of intrahost variants:** Alignment of the high throughput sequence data begins by using the HCMV reference sequence (Merlin, Ref Seq ID: NC_006273). Mismatches between the reference sequence and high throughput sequence reads are identified from the alignment and data about all mismatches (depicted as “C” or “T” in the figure) are outputted into a pileup file. The pileup files is processed with a variant filter protocol that uses threshold values for basecall quality, mapping quality, depth, mismatch frequency, and the number of mismatch occurrences. Mismatches with characteristics above these threshold values are outputted by the variant filter. Mismatches from this filtering are either high frequency (frequency > 0.5, Red “C”) or low frequency (frequency < 0.5, Black “T”). The high frequency mismatches are interpreted to be sample specific polymorphisms, and are incorporated into the sample specific genome type. Additionally, unaligned reads are used to build contiguous sequences (contigs) and are incorporated into the sample specific genome type if showing homology to the sample specific genome type. The sample specific genome type is then used as the reference sequence for additional rounds of alignment of the sequence reads. Again, high frequency polymorphisms are incorporated into the genome type, and contigs are built and assembled onto the genome type. This process is repeated until no additional high throughput sequence reads align to the genome type. The genome type is exported to create the final sample specific genome type (Blue line with incorporated “C” polymorphism). Lastly, the high throughput sequence reads are aligned to final genome type and variants are called to define the intrahost variants of the viral population. For example, the black “T” would be identified as an intrahost variant.

**Figure S5: Scatter plot of ORF Depth vs measured nucleotide diversity:** For all ORFs, the depth from high throughput sequence data is compared to the calculated nucleotide diversity. The red line represents the linear regression through the data. Equation for linear regression: y = (1.153
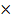
 10^-5^)x + 0.1872. **A.** The plot is shown for all depth values on a logarithmic scale **B.** Same plot as in **A** but only showing depth values between 15-1200, which are the values selected for downstream analysis because the effect of depth on calculated nucleotide diversity is ~.01%.

**Figure S6: HCMV intrahost nucleotide diversity by ORF expression kinetics:** Intrahost nucleotide diversity was calculated for each ORF of the HCMV genome. The ORFs were then grouped by expression kinetics and average nucleotide diversity was calculated across all three patients. Error bars represent the 95% confidence interval for the calculated mean. 1-factor ANOVA test for significance: p = 0.0105 (*not significant after Bonferroni correction*).

**Figure S7: Unrooted phylogenetic trees of clonal Sanger sequencing of HCMV populations:** 3 ORFs per patient sample were selected for clonal Sanger sequencing. The Sanger dataset was then used to generate unrooted phylogenetic trees, using a Jukes-Cantor model of substitution and a neighbor joining method. Scale bars represent substitutions per site. Branch tips are unlabeled except for those representing sequences from the other patient samples, which are highlighted with red text.

**Figure S8: Neutral simulations of HCMV populations:** 1000 simulations of 3 populations using a standard neutral model were generated via the ms program . Theta and the number of segregating sites in each simulation were matched to the corresponding values from the clinical samples U01, U04 and U33. The simulations were then analyzed using the Sweepfinder program and Composite Likelihood Ratios (CLRs) were generated. The CLRs from the simulations were used to calculate significance thresholds. The 5% significance thresholds for each simulation set are shown as red, dotted lines.

**Figure S9: HCMV interhost dN/dS by ORF expression kinetics:** Interhost dN/dS values were calculated for each ORF of the HCMV genome. The ORFs were then grouped by expression kinetics and average nucleotide diversity was calculated across all three patients. Error bars represent the 95% confidence interval for the calculated mean. 1-factor ANOVA test for significance: p = 0.1108.

**Supplemental References**

1. Bale JF, Jr., Petheram SJ, Robertson M, Murph JR, Demmler G (2001) Human cytomegalovirus a sequence and UL144 variability in strains from infected children. J Med Virol 65: 90-96.

2. Bar M, Shannon-Lowe C, Geballe AP (2001) Differentiation of Human Cytomegalovirus Genotypes in Immunocompromised Patients on the Basis of UL4 Gene Polymorphisms. J Infect Dis 183: 218-225.

3. Barbi M, Binda S, Caroppo S, Primache V, Didò P, et al. (2001) CMV gB genotypes and outcome of vertical transmission: study on dried blood spots of congenitally infected babies. Journal of Clinical Virology 21: 75-79.

4. Pignatelli S, Dal Monte P, Rossini G, Lazzarotto T, Gatto MR, et al. (2003) Intrauterine cytomegalovirus infection and glycoprotein N (gN) genotypes. J Clin Virol 28: 38-43.

5. Puchhammer-Stockl E, Gorzer I, Zoufaly A, Jaksch P, Bauer CC, et al. (2006) Emergence of multiple cytomegalovirus strains in blood and lung of lung transplant recipients. Transplantation 81: 187-194.

6. Rasmussen L, Geissler A, Cowan C, Chase A, Winters M (2002) The Genes Encoding the gCIII Complex of Human Cytomegalovirus Exist in Highly Diverse Combinations in Clinical Isolates. J Virol 76: 10841-10848.

7. Sun ZR, Ji YH, Ruan Q, He R, Ma YP, et al. (2009) Structure characterization of human cytomegalovirus UL131A, UL130 and UL128 genes in clinical strains in China. Genet Mol Res 8: 1191-1201.

8. Arav-Boger R, Foster CB, Zong JC, Pass RF (2006) Human cytomegalovirus-encoded alpha -chemokines exhibit high sequence variability in congenitally infected newborns. J Infect Dis 193: 788-791.

9. Heo J, Petheram S, Demmler G, Murph JR, Adler SP, et al. (2008) Polymorphisms within human cytomegalovirus chemokine (UL146/UL147) and cytokine receptor genes (UL144) are not predictive of sequelae in congenitally infected children. Virology 378: 86-96.

10. Arav-Boger R, Willoughby RE, Pass RF, Zong JC, Jang WJ, et al. (2002) Polymorphisms of the cytomegalovirus (CMV)-encoded tumor necrosis factor-alpha and beta-chemokine receptors in congenital CMV disease. J Infect Dis 186: 1057-1064.

11. He R, Ruan Q, Qi Y, Ma YP, Huang YJ, et al. (2006) Sequence variability of human cytomegalovirus UL143 in low-passage clinical isolates. Chin Med J (Engl) 119: 397-402.

12. Ma YP, Ruan Q, He R, Qi Y, Sun ZR, et al. (2006) Sequence variability of the human cytomegalovirus UL141 Open Reading Frame in clinical strains. Arch Virol 151: 827-835.

13. Ji YH, Rong Sun Z, Ruan Q, Guo JJ, He R, et al. (2006) Polymorphisms of human cytomegalovirus UL148A, UL148B, UL148C, UL148D genes in clinical strains. J Clin Virol 37: 252-257.

14. Yan H, Koyano S, Inami Y, Yamamoto Y, Suzutani T, et al. (2008) Genetic variations in the gB, UL144 and UL149 genes of human cytomegalovirus strains collected from congenitally and postnatally infected Japanese children. Arch Virol 153: 667-674.

15. Rasmussen L, Geissler A, Winters M (2003) Inter and Intragenic Variations Complicate the Molecular Epidemiology of Human Cytomegalovirus. The Journal of Infectious Diseases 187: 809-819.

16. Görzer I, Guelly C, Trajanoski S, Puchhammer-Stockl E (2010) Deep sequencing reveals highly complex dynamics of human cytomegalovirus genotypes in transplant patients over time. J Virol: JVI.00475-00410.

17. Hudson RR (2002) Generating samples under a Wright-Fisher neutral model of genetic variation. Bioinformatics 18: 337-338.

18. Nielsen R, Williamson S, Kim Y, Hubisz MJ, Clark AG, et al. (2005) Genomic scans for selective sweeps using SNP data. Genome Res 15: 1566-1575.
